# Correlations of Host and Bacterial Characteristics with Clinical Parameters and Survival in *Staphylococcus aureus* Bacteremia

**DOI:** 10.3390/jcm10071371

**Published:** 2021-03-28

**Authors:** Hannah Wächter, Erdal Yörük, Karsten Becker, Dennis Görlich, Barbara C. Kahl

**Affiliations:** 1 Institute of Medical Microbiology, University Hospital Münster, 48149 Münster, Germany; hannah.waechter@uni-muenster.de (H.W.); erdal.yoe@uni-muenster.de (E.Y.); Karsten.Becker@med.uni-greifswald.de (K.B.); 2 Friedrich Loeffler-Institute of Medical Microbiology, University Medicine Greifswald, 17475 Greifswald, Germany; 3 Institute of Biostatistics and Clinical Research, University Hospital Münster, 48149 Münster, Germany; dennis.goerlich@ukmuenster.de

**Keywords:** *Staphylococcus aureus*, bacteremia, mortality, toxin genes, *agr* type, *spa* type, biofilm, nuclease

## Abstract

*Staphylococcus aureus* bacteremia (SAB) is a frequent, severe condition that occurs in patients of all age groups and affects clinical departments of all medical fields. It is associated with a high mortality rate of 20–30%. In this study, we analyzed patient mortality associated with SAB at our tertiary care university hospital, assessed the clinical management in terms of administered antimicrobial therapy, and determined which factors have an impact on the clinical course and outcome of patients with this disease. We collected clinical data and blood culture isolates of 178 patients diagnosed with SAB between May 2013 and July 2015. For this study, bacteria were cultured and analyzed concerning their phenotype, hemolysis activity, biofilm formation, nuclease activity, prevalence of toxin genes, *spa* and *agr* type. Overall mortality was 24.2% and 30-day mortality was 14.6%. Inadequate initial therapy was administered to 26.2% of patients and was associated with decreased survival (*p* = 0.041). Other factors associated with poor survival were patient age (*p* = 0.003), *agr* type 4 (*p* ≤ 0.001) and pathological leukocyte counts (*p* = 0.029 if elevated and *p* = 0.003 if lowered). The type of infection focus, *spa* clonal complex and enterotoxin genes *seg* and *sei* had an impact on severity of inflammation. Our results indicate that mortality and burden of disease posed by SAB are high at our university hospital.

## 1. Introduction

*Staphylococcus aureus* (*S. aureus*) is one of the most common pathogens to cause a multitude of clinically relevant infections in humans [1,2,3]. Infections range from mild cases of skin or soft tissue colonization to high-risk blood stream infections that are often complicated by the presence of deep foci such as infective endocarditis or other organ disseminations [3]. The reported incidence of *S. aureus* bacteremia (SAB) ranges from 15 to 40 per 100,000 person-years in Western countries [2,4]. In recent years, intensive research has been conducted to reduce SAB morbidity and mortality, and many diagnostic and therapeutic measures have been implemented to improve clinical management, such as standardized antibiotic guidelines, antibiotic stewardship consultation recommendations, or rapid diagnostic testing procedures [3,5,6]. Still, reported SAB mortality rates have failed to decrease substantially and currently range from 20% to 30% even in highly developed medical settings [4,7,8,9,10,11].

In this study, our goal was to analyze why mortality rates remain high despite the exhaustive efforts taken to improve treatment of SAB. Although the impact of host and pathogen features on mortality has been studied extensively and some factors, such as age, presence of comorbidities or persistence of bacteremia for several days have been acknowledged as predictors of worse outcomes [7,8], there is still a lot of uncertainty and heterogenous data concerning the impact of specific bacterial virulence factors on the course and outcome of SAB [7,8]. To address this, we analyzed mortality associated with SAB at our tertiary-care university hospital and examined several clinical and bacterial factors which might have an impact on the clinical course and patient outcome. We retrospectively identified patients diagnosed with SAB at the University Hospital Münster between May 2013 and July 2015, collected clinical data from electronic patient files, and analyzed the bacterial features of *S. aureus* isolates from the first positive blood culture obtained from each patient.

## 2. Materials and Methods

### 2.1. Study Design

Patients were included in this study if they were diagnosed with SAB at the University Hospital Münster, a tertiary-care hospital with more than 1500 beds, between May 2013 and July 2015. For our analysis, we did not identify or exclude patients with possible pseudo-bacteremia. Clinical data were collected retrospectively from electronic patient files and included sex, age, focus of infection, fever, CRP, leukocyte counts, administered antimicrobial agents, survival and mortality. Between one and seven positive blood cultures per patient were collected and frozen at our Institute of Medical Microbiology. For this study, only the earliest *S. aureus*-positive blood culture available from each patient was analyzed (*n* = 178). All isolates were frozen at −80 °C on the date of sampling and thawed for further examination in 2018.

### 2.2. Clinical Data

All clinical data were collected from electronic patient files and medical records using the hospital documentation system ORBIS (Agfa Health Care N.V., Mortsel Belgium). Default settings for the interpretation of laboratory values and inflammation parameters were adopted from ORBIS. Body temperature above 38 °C was defined as fever, CRP was considered elevated if exceeding 0.5 mg/dL and leukocyte counts were considered normal between 3.9 and 10.9 × 10³/µL.

### 2.3. Administered Antimicrobial Therapy

We evaluated the initial therapy that patients received within a week (seven days) after drawing the blood culture. The administered antimicrobial agents were classified as adequate or inadequate based on the guideline for calculated intravenous therapy of bacterial infections from the German Paul Ehrlich Society of Chemotherapy (Paul-Ehrlich Gesellschaft für Chemotherapie) from 2010 [12], which was available at the time the patients in our study were treated (2013 to 2015). This guideline has recently been updated in 2018 with no relevant changes concerning the *S. aureus* treatment recommendations, except for the new inclusion of fosfomycin as a possible combination partner for the treatment of methicillin-susceptible *S. aureus* (MSSA) [5]. Considering that the data about medication that we could retrospectively reconstruct may be partially incomplete, we decided to evaluate the main antibiotic substances only, regardless of dose, therapy duration, resistance profile, infection focus, combination therapy or additional medication applied simultaneously. Antibiotic substances defined as adequate are shown in Table 1. If any of these were administered within the first week after blood culture, treatment was generally considered adequate. In a second analysis, we studied how many patients initially (within the first week) received second-line antibiotics, and in which cases this was appropriate due to a methicillin-resistant *S. aureus* (MRSA) infection. Antibiotics defined as second-line therapy are also shown in Table 1. See Supplementary Table S1 for antibiotic therapy data.

### 2.4. Phenotypic Characterization of Isolates

All isolates were thawed and transferred to Columbia blood agar plates (Becton Dickinson GmbH, Heidelberg, Germany) containing 5% sheep blood and incubated at 37 °C for 24 h. They were transferred to Columbia blood agar plates a second time and incubated at 37 °C for another 24 h, after which the isolates were visually examined for colony size and hemolysis, including beta-toxin positivity. Hemolysis (alpha-toxin) was considered positive if there was a clearing of the agar surrounding the colonies. Isolates were considered positive for beta-toxin in case of a (second) larger and darker hemolysis zone indicative of beta-toxin production if cleared after transfer to 4 °C overnight [13].

The expression of delta-toxin was analyzed via the visible amplification of hemolysis in the interference area of beta- and delta-toxins of different *S. aureus* strains on blood agar plates as described [14]. Briefly, the beta-toxin producing strain RN4220 was transferred to a Columbia blood agar plate in a vertical line and the studied isolates were streaked in horizontal lines reaching close to the RN4220 strain in order to examine the area of interference. In the same way, the positive control RN6607 and the negative control Mu3 were applied. Plates were incubated at 37 °C for 24 h. A strain was considered delta-toxin positive if the area of interference with the RN4220 strain showed an amplified hemolytic activity, i.e., a stronger, larger clearing of the agar. For details on the bacterial strains used in this study, see Appendix A Table A1.

### 2.5. Biofilm Assay

To analyze biofilm formation, we conducted a modified microtiter plate assay that relies on the adherence of biofilm-producing bacteria to smooth surfaces [15]. The method and steps are described in detail elsewhere in the literature [16]. In brief, one colony per isolate was inoculated in 5 mL Brain Heart Infusion (BHI, Difco, Becton Dickinson GmbH, Heidelberg Germany) with 0.25% glucose (BHI + 0.25 g) and grown in a Multitron incubator shaker (Infors Ag, Bottmingen Switzerland) at 160 rpm and 37 °C for 18 h. The overnight culture was diluted in BHI + 0.25 g in a 1:200 ratio and loaded into a 96-well plate which was incubated in a moist chamber at 37 °C for 24 h. The suspension in the wells was discarded and the wells were washed to remove non-adherent bacteria. The remaining biofilm-forming bacteria were stained with crystal violet (Labochem international, neoLab Migge GmbH, Heidelberg Germany), diluted to 0.1%, for 15 min in a dark chamber. Subsequently, the wells were washed again to remove excessive crystal violet. Finally, 100 μL of Ethanol-Acetone (80:20, single reagents purchased from neoFroxx GmbH, Einhausen Germany and Labochem international, neoLab Migge GmbH, Heidelberg Germany, respectively) were transferred into each well to solubilize the remaining stained bacteria. The optical density (OD) of each well was measured at 655 nm in an iMark microplate absorbance reader (Bio-Rad Laboratories GmbH, Feldkirchen Germany) and calculated in percent of the positive control. The cut-off for positive biofilm formation was defined as three standard deviations above the mean of all negative controls. Similarly, the cut-offs for the biofilm categories were calculated by adding three standard deviations of the negative control to the respective previous cut-off. Thus, biofilm formation was considered negative below 4.18% of the positive control, “weak” between 4.18 and 12.52% of the positive control, “moderate” from 12.53 to 37.57% of the positive control, and “strong” from 37.58% of the positive control and above. The assay was conducted in triplicate. A positive control (*S. epidermidis* RP62A), a negative control (*S. carnosus* TM300) and a medium control were used in each new set of plates. For details on the bacterial strains used in this study, see Appendix A Table A1.

### 2.6. Nuclease Assay

The nuclease activity of isolates was analyzed using a fluorescence resonated energy transfer (FRET) assay [17], which was adapted for high-throughput analysis [18]. In brief, isolates were cultured overnight at 37 °C in BHI in a 96-well plate. Growth in each well was documented by measurement of the OD at 578 nm every two minutes in a Synergy HTX multi-mode reader (BioTek Instruments Inc., Winooski, VT, USA). After 16–18 h incubation, the OD was adjusted to OD_578 nm_ = 0.1 (volume 200 µL). The resulting bacterial solution was then incubated for four hours at 37 °C resulting in an OD_578 nm_ = 0.3–0.5. Next, the bacterial supernatant was collected and, in a black 96-well plate positioned on ice, combined with a molecular beacon (10 µM, Sequence 5′→3′: [FAM-CGAATTCC-TTTTT-GGAATTCG-[BHQ1]], Eurofins Genomics Germany GmbH, Ebersberg, Germany), which had previously been suspended in a reaction buffer (50 mM Tris hydrochloride (AppliChem GmbH, Darmstadt, Germany), 5 mM calcium chloride (Sigma-Aldrich Chemie GmbH, Munich, Germany), 100 µg/mL bovine serum albumin (SERVA Electrophoresis GmbH, Heidelberg, Germany), pH 7.9). The fluorescence signal of each well was measured in relative fluorescence units (RFU) in the multi-mode reader with excitation at 485/20 nm, emission at 528/20 nm under fast orbital shaking. The background signal, calculated from the blank control, was subtracted from the sample values and a mean maximal digestion rate (V_Max_) was calculated from the slope of the fluorescence curve of each isolate. The V_Max_ directly represents the nuclease activity of each isolate. The assay was conducted in triplicate. In each new set of plates, two wells were not inoculated with bacteria to represent a blank control, and two wells were inoculated with a positive control known to have a high nuclease activity (*S. aureus* AH1263 wt) and a negative control, in which the *nuc1* gene was knocked out (*S. aureus* AH1263 + Δnuc1 = AH1680), respectively. For details on the bacterial strains used in this study, see Appendix A Table A1.

### 2.7. spa Typing

To characterize the clonality of isolates, we analyzed the highly variable polymorphic X region of the staphylococcal protein A (*spa*) gene by DNA amplification and single locus DNA sequencing as described [19]. For information on the primers used in this study, see Appendix A Table A2. The *spa* type was assigned using the software RidomStaphType (Version 2.2.1, Ridom GmbH, Münster, Germany). The based-upon repeat pattern (BURP) algorithm included in this software grouped related *spa* types into *spa*-clonal complexes (*spa*-CCs) [20]. By default settings, *spa* types shorter than five repeats were excluded. *Spa* types were clustered if cost distances (i.e., evolutionary steps between two *spa* types) were less than or equal to four.

### 2.8. Analysis of Methicillin Resistance, agr Type and Prevalence of Toxin Genes

Single and multiplex PCR were performed to analyze methicillin resistance by determining the *mecA* gene [21], *agr* type [22] and the prevalence of multiple *S. aureus* toxin genes encoding staphylococcal enterotoxins A-E (*sea*, *seb*, *sec*, *sed*, *see*) [23] and G-J (*seg*, *seh*, *sei*, *sej*) [24], toxic shock syndrome toxin (*tst-1*) [23], Panton-Valentine leukocidin (*pvl*) [25], hemolysin gamma (*hlg*) [25], enterotoxins A, B (*eta*, *etb*) [23] and D (*etd*) [26]. The respective primers used for each PCR are given in Appendix A Table A2.

### 2.9. Statistical Analyses

All data were analyzed using the software IBM SPSS Statistics for Windows (IBM Corp. 2017, Version 25.0. Armonk, NY, USA: IBM Corp.). Categorical variables were described by absolute and relative frequencies, while continuous variables were described by either mean or median depending on the distribution of values, which was examined by histogram plots. Standard deviations and interquartile ranges were given as a measure of variation for continuous variables. Categorical variables were compared via Chi-squared tests. Non-parametric tests (e.g., Mann-Whitney U or Kruskal Wallis tests) were used to test for correlations between categorical and continuous variables.

We calculated overall survival as the time from first positive blood culture until last information (dropout or death). Patient mortality was analyzed as relative frequency of patient deaths via crosstabulation and as estimated survival probability in Kaplan Meier analysis. Associations with overall survival (from any cause, censoring at last information) were analyzed using Kaplan Meier survival estimates and Log-Rank tests or Cox-proportional hazard regression. We calculated the median follow-up time via Kaplan Meier estimates with reverse indicator. The influence of inadequate initial therapy on 30-day survival was examined with Kaplan Meier estimates censoring each patient at 30 days. The respective applied statistical test is stated along with each result.

The significance level for all performed tests is 0.05. Given that this is a retrospective study, all statistical testing was of exploratory nature and the results should be confirmed in future prospective studies.

### 2.10. Ethics Statement

This study was approved by the responsible local ethics committee (University of Münster: Ethik-Kommission der Ärztekammer Westfalen-Lippe und der Medizinischen Fakultät der Westfälischen Wilhelms-Universität, registration number: 2018-464-f-S). Patient consent was waived due to the retrospective nature of the study, and the anonymization of patient data.

## 3. Results

### 3.1. Study Cohort and Clinical Data

178 patients diagnosed with SAB at the University Hospital Münster between May 2013 and July 2015 were included in this retrospective study. Clinical data were recovered from electronic patient files and clinical records. Table 2 gives an overview of patient and clinical characteristics.

### 3.2. Administered Antimicrobial Therapy

Data about initial therapy could be evaluated for 145/178 patients, while data of 33 patients were incomplete or missing as reported in Table 2. The adequacy of administered therapy differed depending on methicillin resistance. Data could be evaluated for 132/159 patients with MSSA and 12/18 patients with MRSA bacteremia. Of the patients with MSSA infections, 96/132 (72.7%) initially received adequate treatment. Patients infected by MRSA strains were initially treated appropriately with second-line antibiotics (vancomycin/teicoplanin, daptomycin or linezolid) in 10 out of 12 cases (83.3%), whereas two patients (16.7%) did not receive appropriate second-line therapy within the first week.

### 3.3. Characterization of Isolates

Between one and seven isolates from consecutive positive blood cultures per patient were frozen at our Institute of Medical Microbiology and then thawed and analysed in 2018. For this study, the earliest blood culture available from each patient was examined (*n* = 178). The isolate characteristics are summarized in Table 3.

**Figure 1:**
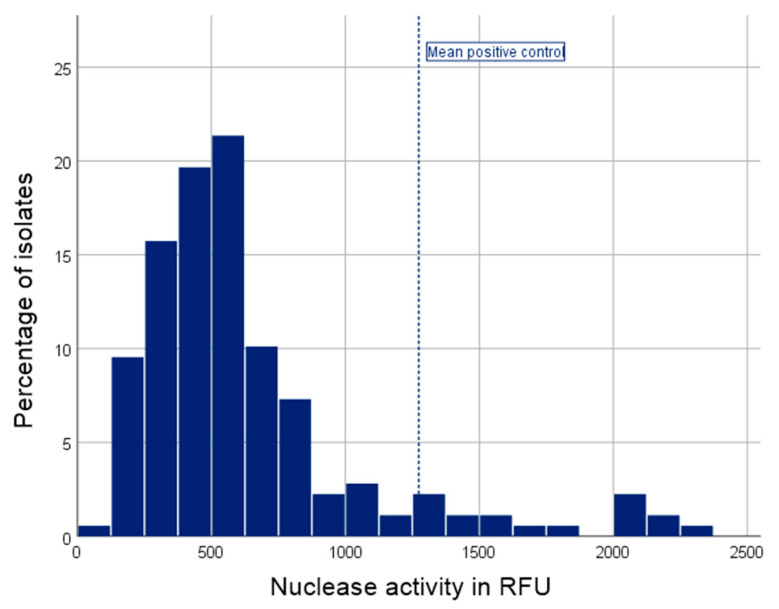
Histogram of the nuclease activity assay results. Nuclease activity was highly variable, but present in all isolates. Median nuclease activity was 528.6 RFU (IQR 358.8–743.9 RFU). The positive control showed a mean nuclease activity of 1274.4 RFU; RFU = relative fluorescence units.

Nuclease activity was highly variable among our clinical SAB isolates (range 97.8–2281.9 RFU, median activity 528.6 RFU). The positive control showed a mean nuclease activity of 1274.4 RFU. The results of the nuclease activity assay are shown in Figure 1.

#### *spa* Typing

A total of 112 different *spa* types were identified within our 178 isolates, most occurring in one or two patients only. Some *spa* types occurred more frequently, such as *spa* types t091 (19 patients, 10.7%), t084 (10 patients, 5.6%), t012 (9 patients, 5.1%), t032 (6 patients, 3.4%), t008 (5 patients, 2.8%), and t026 (4 patients, 2.2%). Two isolates were not typable.

**Figure 2:**
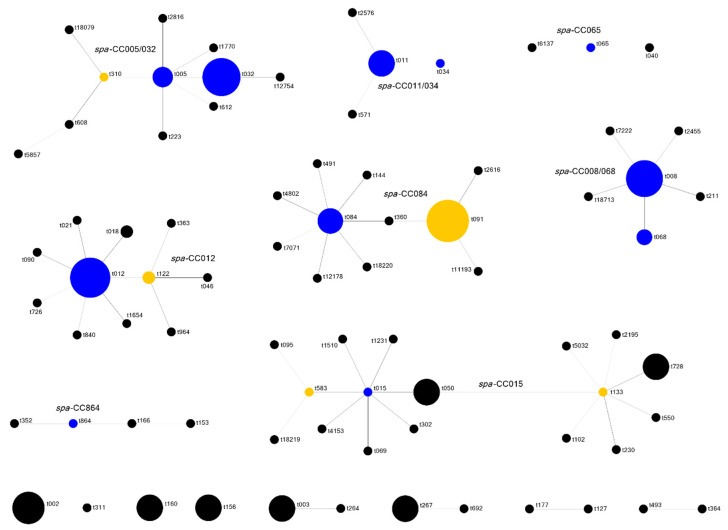
Population structure as analyzed by *spa* typing and BURP clustering. Clonally related *spa* types are grouped together in *spa* clonal complexes (*spa*-CCs). Eight *spa*-CCs comprising 119 isolates were identified. The founder of each complex, i.e., the *spa* type of clonal origin, is calculated by the BURP algorithm as the sum of evolutionary steps between *spa* types which is condensed into founder scores [20]. The *spa* type with the highest founder score is defined as the founder and colored in blue. Should two *spa* types reach the same score, both will be colored in blue. Yellow color indicates the subfounders, identified by the second highest founder score within one complex. The lines connect the founder to the descendant *spa* types of one *spa*-CC, whereby black lines represent a direct relation (one evolutionary step) and lighter shades represent a more distant relation (up to four steps). The size of each circle symbolizes the number of isolates in this study that belong to the respective *spa* type. The size of blank space between different *spa*-CCs was chosen arbitrarily and provides no information about clonal distance between them. 12 *spa* types, comprising 18 isolates, could be grouped into clusters but not complexes as no founder was identified. These are shown at the bottom of this figure. 29 *spa* types were defined as singletons without clonal relation to other *spa* types in this study, these are not depicted in this figure. 10 isolates were excluded from the analysis by default settings as their *spa* sequence was shorter than five repeats. For details on numbers of isolates per *spa* type and *spa*-CC, see Table 4 and Supplementary Table S2.

One-hundred and nineteen isolates (66.9%) were assigned to a *spa*-CC, a total of eight *spa*-CCs was identified. Most common was *spa*-CC084 with 38 assigned isolates (21.3%) of 11 different *spa* types, including the two major *spa* types t091 and t084. Other *spa*-CCs and corresponding *spa* types are shown in Table 4. There was a highly significant association between the *spa* and *agr* type (*p* < 0.0001), and for most *spa*-CCs, all isolates belonged to a single *agr* type. The results of the *spa* typing and BURP analysis are shown in Figure 2, Table 4 and Supplementary Table S2.

### 3.4. Factors with an Impact on Survival and Mortality

#### 3.4.1. Host-Related Factors

**Figure 3:**
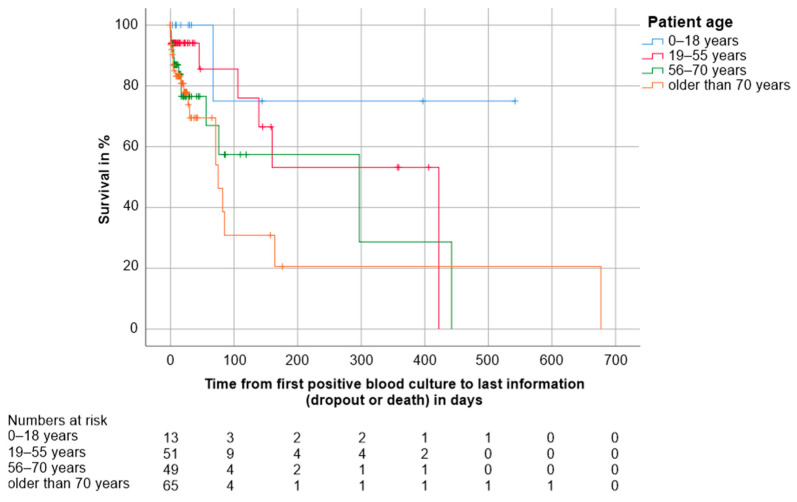
Kaplan Meier survival estimates for the four age groups 0–18 years (*n* = 13, 1 death), 19–55 years (*n* = 51, 8 deaths), 56–70 years (*n* = 49, 13 deaths) and older than 70 years (*n* = 65, 21 deaths). The numbers of patients under observation per age group at each time point are given below the figure. Survival was worse in patients over 70 years compared to those aged 0–18 years (*p* = 0.041) and 19–55 years (*p* = 0.023). Data of three patients were missing.

**Figure 4:**
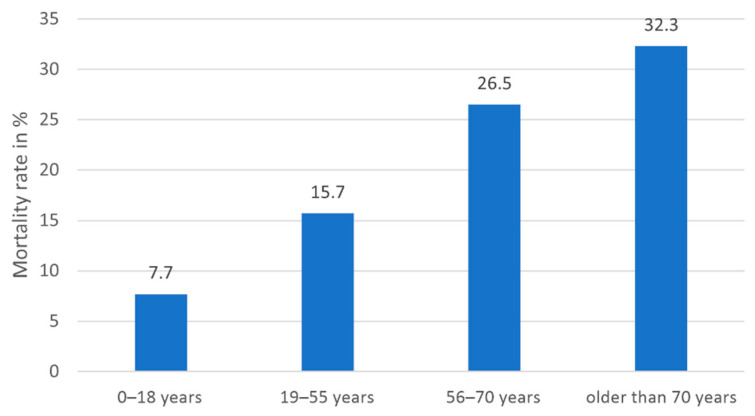
Overall mortality rates in percent per age group. Mortality rates increased with advancing age, whereby survival was worse in patients older than 70 years compared to patients aged 0–18 years (*p* = 0.041) and 19–55 years (*p* = 0.023). Data of three patients were missing.

Patient age. Cox regression revealed a hazard ratio of 1.028 for the influence of patient age on overall survival, indicating that the hazard rate increases by 2.8% with each year (*p* = 0.003, CI 1.010–1.046). The Kaplan-Meier survival analysis showed differences in survival for the four age groups 0–18 years (*n* = 13, 1 death), 19–55 years (*n* = 51, 8 deaths), 56–70 years (*n* = 49, 13 deaths), and older than 70 years (*n* = 65, 21 deaths). Statistically significant were the differences between the age groups of 0–18 years and over 70 years (Log-Rank *p* = 0.041); and 19–55 years compared to over 70 years (Log-Rank *p* = 0.023). For detailed results, see Table 5. The Kaplan-Meier survival estimates are shown in Figure 3. The mortality rates per age group are shown in Figure 4.

Patient sex. The overall mortality rate was 27.0% (*n* = 74, 20 deaths) in women compared to 22.1% (*n* = 104, 23 deaths) in men, 30-day mortality was 16.2% (*n* = 74, 12 deaths) in women and 13.5% (*n* = 104, 14 deaths) in men. These findings were, however, not statistically significant (Pearson Chi-square *p* = 0.451 and *p* = 0.608, respectively).

#### 3.4.2. Pathogen-Related Factors

**Figure 5:**
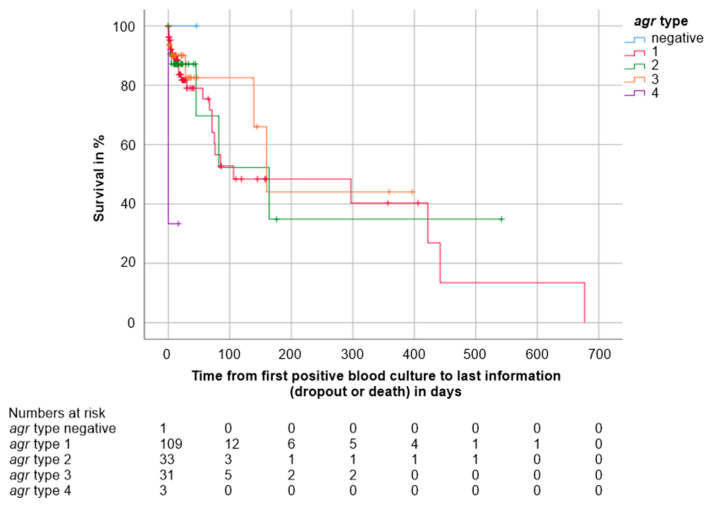
Kaplan Meier survival estimates distinguished by *agr* type: negative (*n* = 1, 0 deaths), *agr* type 1 (*n* = 109, 28 deaths), *agr* type 2 (*n* = 33, 7 deaths), *agr* type 3 (*n* = 31, 6 deaths) and *agr* type 4 (*n* = 3, 2 deaths). The numbers of patients under observation per *agr* type at each time point are given below the figure. Survival was worse in patients infected by isolates of *agr* type 4 compared to all other *agr* types (*p* ≤ 0.001) except for the *agr* negative isolate. Data of one patient were missing.

*agr* type. Kaplan-Meier survival estimates showed that survival of patients infected by isolates of *agr* 4 (*n* = 3, 2 deaths, mortality 66.7%) differed significantly from that of patients infected by isolates of other *agr* types (*agr* 1: *n* = 109, 28 deaths, mortality 25.7%, Log-Rank *p* = 0.0001; *agr* 2: *n* = 33, 7 deaths, mortality 21.2%, Log-Rank *p* = 0.001; *agr* 3: *n* = 31, 6 deaths, mortality 19.4%, Log-Rank *p* = 0.001, missing data of 1 patient). The only non-significant difference was between *agr* 4 and the one isolate negative for all *agr* types (*n* = 1, 0 deaths, Log-Rank *p* = 0.317). For this analysis, no survival time statistics were computed as all cases were censored. Kaplan Meier survival estimates are shown in Figure 5.

#### 3.4.3. Clinical Factors

**Figure 6:**
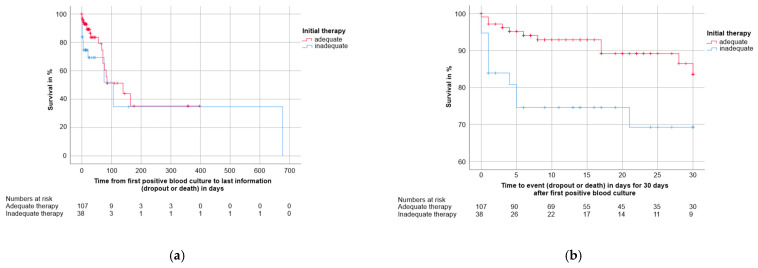
Kaplan-Meier survival estimates distinguished by adequacy of initial therapy. The numbers of patients under observation per therapy group at each time point are given below the figure. Data of 33 patients were missing. (**a**) Overall survival of patients with adequate (*n* = 107, 20 deaths) or inadequate (*n* = 38, 13 deaths) initial therapy. (**b**) 30-day survival of patients with adequate (*n* = 107, 11 deaths) and inadequate (*n* = 38, 10 deaths) initial therapy. Survival was worse in patients with inadequate initial therapy (*p* = 0.041 for (**a**), *p* = 0.010 for (**b**)).

Initial therapy. Kaplan-Meier analysis revealed that overall survival of patients with inadequate initial therapy was worse (*n* = 38, deaths: 13) compared to patients who were initially treated adequately (*n* = 107, deaths: 20, Log-Rank *p* = 0.041, missing data of 33 patients). See Table 6 for detailed results. The survival estimates (shown in Figure 6a) showed an apparent difference between the two groups (patients who were initially treated adequately vs. patients who were initially treated inadequately) especially within the first weeks after drawing the blood culture, suggesting that the highest impact might be on short-term survival. This was confirmed by examining the Kaplan-Meier curves of only the first 30 days (shown in Figure 6b), which revealed an even more obvious difference between the two groups (Log-Rank *p* = 0.010, missing data of 33 patients). See Table 6 for detailed results.

**Figure 7:**
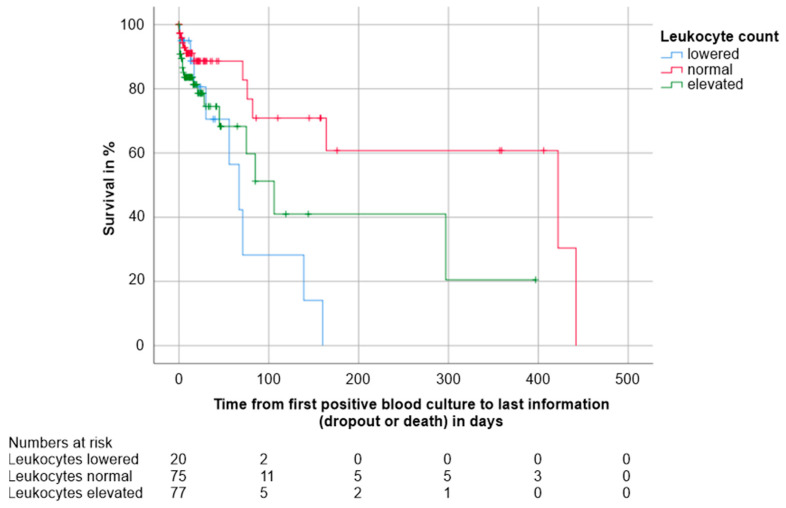
Kaplan-Meier survival estimates distinguished by leukocyte count: lowered, i.e., <3.9 × 10³/µL (*n* = 20, 9 deaths); normal, i.e., 3.9–10.9 x10³/µL (*n* = 75, 13 deaths); and elevated, i.e., >10.9 × 10³/µL (*n* = 77, 20 deaths). The numbers of patients under observation per leukocyte group at each time point are given below the figure. Survival was worse in patients with elevated or lowered leukocyte counts compared to those with normal values (*p* = 0.029 and *p* = 0.003, respectively). Data of six patients were missing.

Leukocyte count. Kaplan-Meier analysis showed that elevated, but especially lowered leukocyte counts were detrimental for patient survival. There were 13 deaths in 75 patients with normal leukocyte values compared to 20 deaths in 77 patients with elevated leukocyte counts and nine deaths in 20 patients with lowered leukocyte counts (Log-Rank of survival curves: elevated vs. normal leukocyte counts *p* = 0.029; lowered vs. normal leukocyte counts *p* = 0.003, missing data of 6 patients). For detailed results, see Table 7. The Kaplan-Meier survival estimates are shown in Figure 7.

Infection focus. Mortality was highest in patients with the infection foci bone and joint infection and pneumonia with 36.4% each (*n* = 11 and *n* = 22, respectively), followed by endocarditis with 31.3% (*n* = 16). It was lowest in abscess and soft tissue infections with 8.7% (*n* = 23) and other, less frequently occurring foci with 0% (*n* = 5). However, none of these differences was statistically significant. Data of one patient were missing in this analysis.

### 3.5. Factors with an Impact on Clinical Parameters and Treatment

#### 3.5.1. Host-Related Factors

Patient age. Patient age was associated with elevated leukocyte levels, as patients with elevated leukocyte levels (*n* = 77) were older (median 66.0 years, IQR 54.5–78.5 years) than patients whose leukocyte levels were within the norm (*n* = 75, median 58.0 years, IQR 44.0–71.0) (Kruskal-Wallis test *p* = 0.014, missing data of 6 patients).

Also, age was associated with increased CRP levels. Patients of the age groups 19–55 years (*n* = 50), 56–70 years (*n* = 46), and above 70 years (*n* = 59) had a significantly higher median CRP value (12.1 mg/dL, IQR 5.5–24.1 mg/dL; 12.4 mg/dL, IQR 9.0–23.2 mg/dL; 17.9 mg/dL, IQR 8.9–24.1 mg/dL; respectively) than patients of the age group 0–18 years (*n* = 11, median 3.3 mg/dL, IQR 1.4–12.8 mg/dL; Kruskal-Wallis tests *p* = 0.011, *p* = 0.005, *p* = 0.001, respectively; missing data of 12 patients).

**Figure 8:**
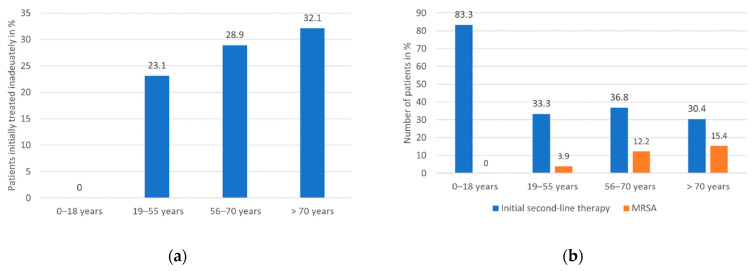
(**a**) Numbers of patients who received inadequate initial therapy per age group in percent. Inadequate initial treatment was associated with advanced age, whereby patients in the age groups 56–70 years and older than 70 years were significantly more likely to receive inadequate initial therapy than patients aged 0–18 years (*p* = 0.035 and *p* = 0.022, respectively; missing data of 33 patients). (**b**) Numbers of patients who initially received second-line therapy or were infected by MRSA in percent. Patients of the age group 0–18 years were likelier to receive second-line antibiotics as initial therapy compared to patients of the age groups 19–55 years (*p* = 0.002), 56–70 years (*p* = 0.005) and older than 70 years (*p* = 0.001, missing data of 33 patients), whereby no patient aged 0–18 years was infected by MRSA.

Age was also associated to inadequate initial therapy, as patients who initially received inadequate therapy (*n* = 38) were significantly older (median 65.5 years, IQR 56.5–79.0 years) compared to those who were treated adequately (*n* = 107, median 63.0 years, IQR 42.0–73.0 years, Mann-Whitney-U test *p* = 0.034, missing data of 33 patients). Furthermore, no patient in the age group 0–18 years (*n* = 12) initially received inadequate treatment compared to nine patients (23.1%) between 19 and 55 years (*n* = 39), 11 patients (28.9%) between 56 and 70 years (*n* = 38) and 18 patients (32.1%) older than 70 years (*n* = 56; Pearson Chi-Square test: 0–18 years vs. 19–55 years *p* = 0.067; 0–18 years vs. 56–70 years *p* = 0.035; 0–18 years vs. older than 70 years *p* = 0.022, missing data of 33 patients). The numbers of patients per age group initially treated with inadequate therapy are depicted in Figure 8a.

The initial administration of second-line antibiotics was also associated with age. Patients who received second-line antibiotics within the first week of treatment (*n* = 54) were younger (median 59.5 years, IQR 38.0–73.0 years) than patients who received such antibiotics later or not at all (*n* = 91, median 65.0 years, IQR 52.0–78.0 years; Mann-Whitney-U test *p* = 0.023). Furthermore, patients of the age group 0–18 years were significantly more likely to receive second-line antibiotics as initial therapy than patients of all other age groups (administration of second-line antibiotics per age group: 0–18 years: 83.3% of patients (*n* = 12), 19–55 years: 33.3% of patients (*n* = 39), 56–70 years: 36.8% of patients (*n* = 38), older than 70 years: 30.4% of patients (*n* = 56; Pearson Chi-Square test: 0–18 years vs. 19–55 years *p* = 0.002, 0–18 years vs. 56–70 years *p* = 0.005, 0–18 years vs. older than 70 years *p* = 0.001), missing data of 33 patients). The numbers of patients per age group initially treated with second-line antibiotics are depicted in Figure 8b.

Age was also associated with methicillin resistance, as patients infected by MRSA (*n* = 18) were significantly older (median 73.5 years, IQR 58.5–78.0 years) than patients infected by MSSA (*n* = 159, median 62 years, IQR 49.0–73.0 years; Mann-Whitney-U test *p* = 0.037, missing data of 1 patient). The MRSA frequency in the four age groups was as follows: 0–18 years: 0/12 patients, 19–55 years: 2/51 patients (3.9%), 56–70 years: 6/49 patients (12.2%), older than 70 years: 10/65 patients (15.4%). The numbers of patients per age group infected by MRSA are depicted in Figure 8b.

#### 3.5.2. Pathogen-Related Factors

S*pa*-CC. Some *spa*-CCs were associated with clinical parameters in terms of leukocyte counts. While the median overall leukocyte value was 10.2 × 10³/µL, patients infected by isolates of *spa*-CCs 084 (*n* = 36) and 864 (*n* = 4) had higher leukocyte counts (medians 12.9 and 16.4 × 10³/µL, IQRs 9.0–17.8 and 12.9–27.9 × 10³/µL, Mann-Whitney-U tests *p* = 0.004 and *p* = 0.045, respectively), whereas patients infected by isolates of *spa*-CC068/008 (*n* = 11) had lower leukocyte counts (median 7.4 × 10³/µL, IQR 3.4–11.2 × 10³/µL, Mann-Whitney-U test *p* = 0.025, missing data of 8 patients).

*Seg*/*sei*. Patients infected by *seg* or *sei* positive isolates were more likely to experience fever than patients infected by *seg* or *sei* negative isolates (*seg* positive: fever in 71/91 = 78.0% of patients, *seg* negative: fever in 40/64 = 62.5% of patients, Pearson Chi-Square *p* = 0.035; *sei* positive: fever in 72/92 = 78.3% of patients, *sei* negative: fever in 39/63 = 61.9% of patients, Pearson Chi-Square *p* = 0.027; missing data of 23 patients).

#### 3.5.3. Host-Pathogen Interactions

Infection focus. The type of infection focus was associated with the presence of fever and CRP levels. While fever occurred in 71.8% of patients overall, it occurred more frequently in patients with the focus intravascular device (57/66 = 86.4% of patients, Pearson Chi-Square *p* = 0.001) and was rare in patients with the focus bone and joint infection (4/10 = 40.0% of patients, Pearson Chi-Square *p* = 0.021, missing data of 23 patients). While the median overall CRP value was 13.6 mg/dL, bone and joint-related infections (focus bone and joint implant (*n* = 10) and focus bone and joint infection (*n* = 11)) showed significantly higher CRP values (medians 23.4 and 27.9 mg/dL, IQRs 17.6–29.6 and 11.7–32.1 mg/dL, Mann-Whitney-U test *p* = 0.009 and *p* = 0.011, respectively), whereas CRP values were significantly lower in the infection focus intravascular device (*n* = 62, median 9.0 mg/dL, IQR 4.2–19.1 mg/dL, Mann-Whitney-U test *p* = 0.001) and in patients where the infection focus remained unclear (*n* = 16, median 8.5 mg/dL, IQR 4.2–14.6 mg/dL, Mann-Whitney-U test *p* = 0.034, missing data of 12 patients).

### 3.6. Correlations between Pathogen Features

MRSA. Methicillin resistance was associated to beta-toxin production, which was observed in 8/18 = 44.4% of MRSA isolates compared to 14/159 = 8.8% of MSSA isolates (Pearson Chi-Square *p* = 0.0001, missing data of 1 patient). Beta-toxin production in MRSA isolates was significantly associated with *spa*-CC 034/011 (Pearson Chi-Square *p* = 0.0001, missing data of 2 patients), which comprised four beta-toxin producing isolates of *spa* types t011, t034 and t2576. One isolate belonged to *spa*-CC 005/032 and one to *spa*-CC 068/008, the two remaining isolates were not associated to a *spa*-CC. Methicillin resistance was also correlated with the prevalence of enterotoxin gene *sec*. MRSA isolates were *sec* positive in 7/18 = 38.9% of cases compared to 30/159 = 18.9% of MSSA isolates (Pearson Chi-Square *p* = 0.048, missing data of 1 patient).

Biofilm formation. The formation of biofilm was also associated with the presence of *sec*, as 8/21 = 38.1% of biofilm-forming isolates were *sec* positive vs. 29/156 = 18.6% of biofilm negative isolates (Pearson Chi-Square *p* = 0.039, missing data of 1 patient). All isolates that produced a moderate or strong biofilm (*n* = 3) were *sec* positive. Biofilm formation was also correlated with *spa*-CC068/008. Biofilm was produced by 4/11 = 36.36% of *spa*-CC068/008 isolates, compared to 17/167 = 10.2% of other isolates (Pearson Chi-Square *p* = 0.009, missing data of 1 patient). In detail, two isolates of *spa* type t008 and the isolate of *spa* type t211 produced a weak biofilm, while the isolate of *spa* type t2455 produced a moderate biofilm. The only SCV isolate was simultaneously the only isolate to produce a strong biofilm (Pearson Chi-Square *p* < 0.0001).

Nuclease activity. Isolates obtained from women (*n* = 74) presented with a higher nuclease activity (median 575.0 RFU, IQR 450.7–793.0 RFU) compared to those obtained from men (*n* = 104, median 455.0 RFU, IQR 326.3–683.7 RFU; Mann-Whitney-U *p* = 0.008).

Delta-toxin. The production of delta-toxin was inversely associated with the secretion of beta-toxin, as most isolates produced either delta-toxin (86 isolates) or beta-toxin (18 isolates), only one isolate produced both toxins (Pearson Chi-Square *p* = 0.0001). Some *spa*-CCs were associated with decreased delta-toxin production, which occurred in 48.9% of all isolates, but was rare in isolates of *spa*-CC012 (2/21 = 9.5% of isolates, Pearson Chi-Square *p* = 0.0001) and *spa*-CC864 (0/4 isolates, Pearson Chi-Square *p* = 0.048, missing data of 2 patients). Most isolates of *spa*-CCs 012 and 864 belonged to *agr* type 3, to which delta-toxin also showed an inverse association. Only 2/31 = 6.5% of *agr* 3 isolates produced delta-toxin, compared to 85/146 = 58.2% of other isolates (Pearson Chi-Square *p* < 0.0001, missing data of 1 patient).

*tst-1*. *Spa*-CCs 012 and 864 showed increased prevalence of *tst-1*, which was present in 12.9% of all isolates, but more common in *spa*-CC012 (10/21 = 47.6% of isolates, Pearson Chi-Square *p* < 0.0001) and *spa*-CC864 (4/4 = 100% of isolates, Pearson Chi-Square *p* < 0.0001), missing data of 3 patients). *Tst-1* was also correlated with *agr* type 3. Isolates of *agr* 3 were significantly more often *tst-1* positive (15/31 = 48.4%) than other isolates (8/146 = 5.5%) (Pearson Chi-Square *p* < 0.0001, missing data of 1 patient). Accordingly, *tst-1* and delta-toxin were inversely associated. *tst-1* positive isolates produced delta-toxin in 4/23 = 17.4% of cases compared to 83/154 = 53.9% of *tst-1* negative isolates (Pearson Chi-Square *p* = 0.001, missing data of 1 patient).

## 4. Discussion

We conducted a retrospective study involving 178 patients diagnosed with SAB at the University Hospital Münster to analyze the mortality and treatment of this disease at our clinic and to identify factors associated with patient outcome and disease severity. Furthermore, we used the stored isolates to perform various phenotypical and genotypical tests to analyze correlations between host and bacterial characteristics. These analyses revealed several interesting results.

### 4.1. Demographic and Clinical Data

The baseline demographic structure of our study population (median age 63.0 years, 58.4% males) resembles that of other clinical studies on SAB [8,9].

Overall all-cause mortality (24.2%) and 30-day mortality (14.6%) range within the reported mortality rates in the literature of 20–30% overall [4,7,8,9,10] and show that even in our highly developed medical setting of a university hospital, SAB remains a substantial threat to patient survival.

Increasing patient age was the parameter with the most associations to outcome, clinical course, and pathogen features. It was significantly associated with decreased survival, and mortality rates were highest in patients older than 70 years, which is in line with literature findings that describe advanced patient age as the most consistent predictor of mortality in SAB [3,7,27,28]. Old age was also correlated with enhanced disease severity in terms of higher leukocyte and CRP levels in line with earlier literature [27]. Also, methicillin resistance was more frequent in the elderly, which is likely due to a higher rate of healthcare-associated infections in long-term care units and nursing homes as described recently [29,30,31]. Conversely, increasing age was also associated with the administration of inadequate initial therapy in this present study. Despite a greater risk of aggravated infection and poorer outcome, almost one third of patients older than 70 years received inadequate antibiotics in the first week of treatment, which is an alarming finding and highlights the need for improvement of therapeutic management in elderly patients. Also, the application of initial second-line treatment was significantly less common in elderly than in younger patients, although the distribution of MRSA was vice-versa. Pediatric patients received second-line antibiotics in 83.3% of cases, although not a single pediatric patient was infected by MRSA.

The most common focus of infection in our study was an intravascular device (39.9% of patients), followed by abscess and soft tissue infections (12.9%), pneumonia (12.4%) and endocarditis (9.0%). These findings are in line with clinical data from other hospitals, where intravenous catheters and skin and soft tissue infections are often reported as the most frequently found focus of SAB [8,9,11]. The infection focus is described as one of the major predictors of mortality in SAB, whereby mortality rates are highest in pneumonia and endocarditis [7,8,28]. We report highest mortality rates in the foci bone and joint infection and pneumonia, followed closely by endocarditis. However, these differences in mortality were not statistically significant, possibly due to an insufficient number of patients in each focus group (*n* = 11 in bone and joint infections, *n* = 22 in pneumonia, *n* = 16 in endocarditis). The type of infection focus was significantly associated with clinical parameters: while patients with intravascular device-related bacteremia were more likely to experience fever but had lower CRP levels, patients with bone and joint-focused SAB presented with less fever but higher CRP levels. SAB with deep-seated foci such as osteomyelitis has been associated to higher CRP levels compared to intravenous catheter-related infections before [32], while the inverse dynamic of fever is an interesting additional finding for which we found no previous evidence.

In total, 71.8% of patients presented with fever; 95.8% with elevated CRP levels; 44.8% with elevated and 11.6% with lowered leukocyte counts. These data are not generally reported in studies on SAB, but in the few others that do report these parameters, similar results are described [27]. Less than half of all patients presented with leukocytosis, which is especially interesting as it is often assumed to be a clinical feature of bacteremia and sepsis and used to assess the clinical course of the disease. Some clinical parameters were associated with mortality, host and pathogen features. Most importantly, we report decreased survival in patients with elevated, but especially in those with lowered leukocyte counts. Leukocytosis, especially if unresolving, has been described to be correlated with mortality in other studies before [33], although it is not one of the major predictors of mortality in SAB. Immunosuppression or immune deficiency per se has been reported to have a detrimental effect on patient outcome in SAB [7,34], however, it is unclear if the leukopenia that occurred in 20 patients in our study was due to underlying immune deficiency or a result of the bacteremia itself. Still, our results suggest an important role of leukocyte counts as possible early clinical predictors of patient outcome in SAB, which should be further investigated in future prospective studies.

Another correlation of clinical parameters was with clonality of isolates and prevalence of certain toxins. Leukocyte counts were higher in patients infected by *spa*-CCs 084 and 864, and lower in those infected by *spa*-CC068/008, which also showed an increased biofilm production. These findings deliver interesting hints toward the different strategies adopted by *S. aureus* clones to overcome host defense mechanisms, following recent evidence that not all *S. aureus* strains achieve virulence and invasiveness in the same way [35]. Furthermore, fever was significantly associated with the prevalence of *seg* and *sei*, which is possibly due to these enterotoxins’ role as superantigens and potent T-cell-activators that lead to acute inflammation responses [36,37].

### 4.2. Administered Antimicrobial Therapy

A surprisingly high rate of 26.2% of all patients did not receive antibiotics classified as generally adequate for SAB within the first week of treatment; for patients infected by MSSA, it was even 27.3%. Patients with MRSA bacteremia were inadequately treated (without second- line antibiotics) in 16.7% of cases, showing that correct treatment was more likely in case of MRSA than MSSA bacteremic infections. A possible reason for this might be the serious attention paid to MRSA as a hard-to-eradicate hospital pathogen, leading to an increase in importance attributed to MRSA infections and an improvement in treatment accuracy. Since it remains unclear if MRSA as a cause of bacteremia is associated with increased mortality [7], this might partly be warranted; although MRSA was no independent factor correlated with poor patient outcomes in our present study.

29.7% of patients wrongly received second-line antibiotics for MSSA infections in the first week of treatment, whereby this rate was particularly high in the age group of 0–18 year-olds with 83.3% (10 out of 12 patients). This needs to be seen very critically especially in the context of increasing antibiotic resistance in pathogens world-wide, which may be aggravated or accelerated by the irrational use of broad-spectrum or second-line antibiotics for infections with not-yet-resistant pathogens.

The number of patients that received inadequate initial therapy and that of patients with unnecessary second-line treatment was alarmingly high in our tertiary care university hospital, especially when considering that inadequate initial treatment was significantly associated with decreased survival in our present study, and is also described as a main predictor for patient mortality in the literature [7,28,38]. However, it should be noted that the time frame studied here (2013 to 2015) was before a standardized antibiotic stewardship consultation was implemented in our hospital, so it can be assumed that the antimicrobial treatment has improved by now as could be shown by several studies in the literature [6,7,34,39,40]. Still, our results highlight that the correct and quick application of initial calculated antibiotic therapy is of fundamental importance for patient survival and should be handled accordingly by the attending physician. As discussed above, especially elderly patients seem to be under great risk of receiving inadequate therapy and should be treated with utmost attention.

### 4.3. Colony Size and Hemolysis Activity

All isolates were visually analyzed for colony size and the activity of hemolytic toxins. Colony size was normal in almost all isolates (97.8%), only one isolate (0.6%) was identified as an SCV. Since SCVs have been shown to be associated with chronic, persistent and recurrent rather than acute infections [41,42], this result was to be expected, as the isolates studied here were taken from the earliest available blood cultures of patients with SAB and thus represent the early stages of the disease. Three isolates (1.7%) showed mixed colony growth on blood agar plates, with some SCVs and some normal-sized colonies, which could be due to a focus that persisted already for some time.

Alpha-toxin, a pore-forming exotoxin capable of cell lysis and endothelial disruption, is a major virulence factor that is produced by most *S. aureus* strains [43,44,45,46,47]. The vast majority of isolates in our study (98.3%) showed hemolytic alpha-toxin activity, confirming how well-conserved this toxin is in *S. aureus* and validating its role as a possible target for selective anti-staphylococcal treatment [46,48].

Beta-toxin is a sphingomyelinase that targets immune cells and erythrocytes [49,50] and is encoded by virtually all *S. aureus* strains but secreted by only a small fraction, as the *hlb* gene is deactivated by a bacteriophage in most *S. aureus* strains [51,52,53,54]. Accordingly, only 19 of our isolates (10.7%) exhibited beta-toxin production, in line with the reported prevalence in the literature of around 13% [55]. Another three isolates (1.7%) showed heterogenous behavior with beta-toxin secretion only around some colonies, which could be due to partial bacteriophage excision in these isolates.

In our statistical analysis, we found a significant association between in vitro beta-toxin production and methicillin resistance. MRSA isolates exhibited beta-toxin production in 44.4% of cases, compared to only 8.8% in MSSA. This is a very interesting finding, as it indicates that carriage of the bacteriophage responsible for the lack of beta-toxin production in most *S. aureus* isolates might be rarer in MRSA strains. To further investigate this, we analyzed the clonality of beta-toxin producing MRSA isolates and found a significant correlation to *spa*-CC 034/011. The corresponding *spa* types t011, t034 and t2576 are also indicative of LA-MRSA. We hereby confirmed a link between beta-toxin production (and therefore lack of the deactivating bacteriophage) and livestock association, which matches the results of previous studies that reported intact beta toxin in the majority of *S. aureus* isolates from livestock [55,56,57].

Delta-toxin is used as a surrogate marker for the activity of the *agr* locus, a main virulence-regulating system of *S. aureus* [50,58,59,60,61], and lack of delta-toxin production is regarded as a characteristic of *agr*-inactive *S. aureus* strains [60,61]. Overall, only 48.9% of our isolates exhibited delta-toxin activity. However, this rate might be higher in vivo than in our in vitro study environment, as *agr* function is labile under in vitro conditions [14].

The lack of delta-toxin expression, and therefore *agr* deficiency, was significantly associated with a variety of pathogen features in our study. First, we report an inverse correlation between delta-toxin and beta-toxin secretion, as all studied isolates but one expressed either one toxin, but not both simultaneously. We also found an inverse correlation between delta-toxin and *tst-1*, which seems to be an effect of clonal origin and genetic differences in toxin prevalence. Delta-toxin expression was rare in *spa*-CCs 012 and 864, which mainly belonged to *agr* type 3. Accordingly, delta-toxin expression also showed an inverse correlation with *agr* type 3. At the same time, *spa*-CCs 012 and 864 displayed enhanced prevalence of *tst-1*, which, in turn, was positively associated with *agr* type 3.

### 4.4. Biofilm

Most studies on biofilm formation of invasive clinical *S. aureus* isolates report that the majority of isolates were capable of biofilm formation [62,63,64]. Conversely, biofilm formation occurred in only 11.8% of isolates in our study, whereby most of these isolates produced only a weak biofilm. It has been hypothesized that the transformation from a planktonic to a sessile, biofilm-producing state is a feature of more chronic and persistent infections [65,66,67], and might even decrease pathogen virulence and hinder the establishment of invasive infection by initial spread in the blood stream [66]. Therefore, a low number of biofilm formers in patients with acute onset of SAB is not an unexpected finding. In contrast to another study, which showed that an inactive *agr*-locus of *S. aureus* is associated with increased biofilm formation [68], the delta-toxin negative isolates in our study did not produce more biofilm compared to the delta-toxin positive isolates. Since we used comparable methods for delta-toxin activity and biofilm formation, such a difference in biofilm formation could be probably due to the different genetic backgrounds of strains in the US and in our area, because in the US the prevalence of MRSA strains is much higher compared to our area. Unfortunately, in the study of Vuong et al., there is no information about the investigated strains.

Another factor that might explain the low number of biofilm-forming isolates in our study is the high level of nuclease activity that was found in most isolates. The secreted *S. aureus* enzyme nuclease cleaves DNA molecules and is crucial for *S. aureus* defense against human neutrophils, but has also been shown to inhibit the formation of biofilm or induce the dispersal of existing biofilms [67,69,70,71,72], as *S. aureus* biofilms partly consist of DNA [71]. Therefore, an inverse correlation between secretion of nuclease and biofilm formation has been reported [69]. In our isolates, no statistically significant correlation between nuclease and biofilm formation could be found, possibly due to an insufficient sample size of only 21 biofilm-forming isolates, of which 18 only produced a weak biofilm.

Biofilm formation showed a statistically significant association with the staphylococcal enterotoxin C gene (*sec*), *spa*-CC068/008, and SCV. To our knowledge, the association between biofilm and *sec* is reported here for the first time and is especially interesting as we also report an association of *sec* and methicillin resistance. In a recent, so far preliminary study, *sec* has been described as an inhibitor of endothelial cell migration and vascular repair, and a crucial contributor to infective endocarditis [73], making it an interesting subject for future research. Associations of staphylococcal biofilm to clonal lineage, albeit not specifically *spa*-CC068/008, have been described before [64]. The formation of SCV and biofilm both contribute to the adaptation of *S. aureus* to environmental stress during persistent infection [42,65,66,74]. It has been shown that some strains either convert to SCV or produce biofilm, while others achieve both simultaneously [74]. The latter was the case in our present study, as the one SCV isolate was at the same time the only isolate to produce a strong biofilm.

There was no correlation between biofilm production and disease severity or patient survival in our present study, which is in line with findings in the literature [62,63]. We found no association between biofilm production and infection focus either, which is confirmed by some studies in the literature [64], although others report such a correlation [62].

### 4.5. Nuclease

The enzyme nuclease is recognized as an important *S. aureus* virulence factor that contributes to the establishment of invasive disease [18,75,76] by facilitating evasion from the host immune system in the form of neutrophil extracellular traps (NETs) [75] and dispersing bacterial biofilm, thereby promoting the spread of bacteria to different sites [72]. Accordingly, all of our invasive bacteremia isolates showed nuclease expression, whereby the nuclease activity level was highly variable, and some isolates presented with an outstandingly high nuclease activity. We found no associations between the level of nuclease activity and other pathogen characteristics, and there were no correlations with patient outcome or clinical features. The only exception is an association with female sex, revealed by a significantly higher nuclease activity in blood cultures obtained from female compared to male patients. This is a very interesting finding that, to our knowledge, has not been described before. Data on sexual dimorphisms in bacterial infections are generally scarce, but there is some evidence that, besides behavioural differences that may influence the risk of colonization, infection and outcome, there may also be underlying physiological, immunological or hormonal differences in males and females that affect the host pathogen interplay [77]. Neither the details on these differences nor their exact influence on the interaction between human immune response and bacterium have yet been understood. While it has been generally recognized that males are at a higher risk of infection with SAB [8,77,78], there is evidence for an increased mortality rate in females [7,27,28,38,77,79]. Both findings correspond to the data in our present study, although the higher mortality rate in women was not statistically significant. However, a link between the worse outcome in females and the reported increased nuclease activity is conceivable and would certainly be a worthwhile subject for future prospective research.

### 4.6. Clonality

The most common *spa* types found in our isolates were t091 (10.7%), t084 (5.6%) and t012 (5.1%). Eight *spa*-CCs were identified, the most prevalent being *spa*-CC084 (21.3%), followed by *spa*-CC012 (11.8%), *spa*-CC015 (10.7%) and *spa*-CC005/032 (10.2%). The prevalence of *spa* types and *spa*-CCs and therefore the clonality of *S. aureus* strains recovered from invasive infections varies greatly between different regions [80]. Therefore, these results provide a good overview of the epidemiological background of invasive *S. aureus* strains in our geographical area and match those of a study on *S. aureus* clonality in nasal swabs of the general population in the region of Münster from 2017 [81]. An interesting aspect for future studies on *S. aureus* at our hospital will be the comparison of future *spa* types with our reported results, as significant changes in clonal population structure over time have been documented [81,82].

We report no associations of *S. aureus spa* type or -CC with patient survival or mortality, which is in line with literature findings as clonality alone is not described as a predictor for mortality in SAB [7,82], although there is evidence that different pathogen features can influence patient outcome depending on the clonal background [35].

However, some *spa*-CCs were correlated with clinical parameters or pathogen characteristics: *spa*-CCs 084 and 864 with higher-than-average leukocyte counts, *spa*-CC068/008 with low leukocyte counts and increased biofilm production, and *spa*-CCs 012 and 864 with low delta-toxin activity and high prevalence of the *tst* gene.

### 4.7. Methicillin Resistance

In our cohort of invasive SAB isolates, 10.2% of isolates were MRSA, which roughly matches the incidence in Germany in 2015 [83]. While methicillin resistance has been associated with increased mortality in many studies [7,8], we did not observe such a correlation. Methicillin resistance was significantly associated with increasing patient age, expression of beta-toxin and prevalence of *sec*.

The increasing use of antibiotics in livestock farming has led to the rise of LA-MRSA that was shown to have high rates of nasal colonization in farmers and is capable of causing invasive infections [84,85]. Most LA-MRSA *spa* types belong to MLST CC398 [84,85,86,87], of which t011, t034 and t2576 were also found in this present study. Four out of 18 MRSA isolates from SAB (22.2%) could be related to LA-MRSA, an outstandingly high rate compared to the average rate of 1.7% in MRSA from septicemia in North Rhine-Westphalia [88], which is similar to the average rate in all of Germany [89]. The region of Münster and its surrounding districts are rural areas with a very high rate of pig production, and higher-than-average LA-MRSA rates in bacteremia isolates of 6–10% in and around Münster and >11% in the adjacent district of Steinfurt have been described earlier [88]. In 2013, the rate of LA-MRSA among all MRSA infections at our hospital was 35% [90]. Our findings confirm the importance of livestock farms as a reservoir for MRSA in our geographical region and demonstrate the invasive potential of these isolates.

### 4.8. agr Type and Toxin Gene Prevalence

The secretion of *S. aureus* virulence factors including secreted toxins and surface proteins is mostly regulated by the *agr* system, which is initiated via an auto-induced peptide that is variable between *S. aureus* strains [14,91]. Four variations have been described and thereby, four *agr* types have been defined and designated *agr* 1–4 [91,92]. The most prevalent *agr* type with 61.6% of isolates was *agr* 1, followed by *agr* 2 (18.6%), *agr* 3 (17.5%) and *agr* 4 (1.7%). One isolate tested negative for all four *agr* types. Although *agr* defective *S. aureus* strains have been described before [14], a whole-genome analysis would be required to finally classify this isolate as *agr* negative. The distribution of *S. aureus* strains among the four *agr* types varies depending on the geographical region [14,22,93], as *agr* types are strongly linked to the genetic background of isolates [93], which is confirmed by a strong association between *spa* and *agr* type found in our present study. However, most studies report *agr* 1 as the most frequently found *agr* type, while *agr* 4 is found only very rarely [7,14,22]. While there are heterogenous results concerning the influence of *agr* type on mortality and disease severity [7,28], we report decreased survival in patients infected by isolates of *agr* 4. Despite the small sample size of only three isolates, this result was statistically significant. We recently reported a link between *agr* 4 and increased rates of disease exacerbation in *S. aureus* infections in cystic fibrosis [94], another hint toward *agr* 4 aggressiveness in invasive infection. Considering the low prevalence of *agr* type 4, a large-scale prospective study would be necessary to confirm this finding.

As expected, most of the toxin genes were rarely found in our isolates, mostly in less than 10% of cases. An obvious exception is *hlg*, the gene encoding hemolysin gamma, a leukotoxin capable of lysing neutrophils and erythrocytes that is highly preserved and ubiquitously present in *S. aureus* [25,95]. Accordingly, *hlg* was found in all isolates except one. Second most prevalent was the combination of *seg*/*sei* in 58.2% of isolates which is line with findings in earlier studies [24,25]; 20.9% of isolates were *sec* positive and *tst-1* was present in 13.0% of isolates, which is in the range of prevalence rates described in the literature [24,96].

In our present study, enterotoxin genes *seg* and *sei* were associated with the development of fever in patients infected by strains carrying these genes, and *sec* was associated with increased biofilm production and methicillin resistance. These results reveal that analysis of toxin genes can provide interesting information about clinically relevant features of *S. aureus* strains and should be conducted more frequently in studies on SAB.

### 4.9. Study Limitations and Strengths

This study has some limitations. It is a retrospective study, and all clinical data were collected from existing patient files, so only data that had been recorded at the time of treatment could be retrieved. Therefore, information on some interesting aspects were missing or incomplete and could not be evaluated, including patient comorbidities, which are known to have an influence on clinical course and mortality of SAB [7]; and information on the onset of infection (hospital or community acquired), which makes a difference in terms of pathogen features and outcome [3,8]. Due to the lack of data on comorbidities, no confounder-adjusted analyses could be conducted; and we could not examine disease-specific attributable mortality but only overall all-cause mortality. Data on antibiotic therapy may be partially incomplete, so we only analyzed the main antibiotic agents without regard to dose, therapy duration, or combination therapy; and may have thus overestimated the number of patients with adequate initial treatment. Also, some of our results might be weakened by the fact that we did not identify and exclude patients with pseudo-bacteremia due to contamination with *S. aureus*. Data on the laboratory value procalcitonin, which is important in diagnosing and monitoring bacteremia and sepsis, was only available for a few patients and was therefore not evaluated. Follow-up time was relatively short in most patients, the median follow-up time being 21.0 days. This is also due to this study’s retrospective nature, as we could not schedule fixed follow-up visits as in prospective studies. This causes the low numbers at risk in the Kaplan Meier survival estimates, which decrease rapidly after 30 days; and might have led to an underestimated long-term mortality. Furthermore, this study was a single-center study over the course of two years that included 178 patients, which is a considerable number, but is too small to study rare pathogen characteristics (e.g., *agr* type 4) in detail and generate conclusive results. Finally, all statistical testing could not be planned in foresight but had to be conducted retrospectively. Therefore, it was of exploratory nature, and all findings should be confirmed in future prospective studies.

This study’s greatest strength is the multitude of factors including clinical parameters, pathogen features, host characteristics and even administered antibiotic therapy which have all been analyzed in terms of their association with mortality, disease severity, and with one another. While most studies, except reviews and meta-analyses, concentrate on either clinical or microbiological aspects of SAB, we aimed at gaining a wholistic view on this disease, how it is handled at our hospital, and which factors drive its invasiveness and virulence.

## 5. Conclusions

Mortality associated with SAB was high at our university hospital with an overall all-cause mortality of 24.2% and 30-day mortality of 14.6%. Increasing patient age was the parameter with the most associations to outcome, clinical course, and pathogen features. It was significantly associated with decreased survival, worse clinical parameters in terms of higher leukocyte and CRP levels, inadequate initial therapy, methicillin resistance, and rarer application of initial second-line treatment. Besides patient age, poor survival was also associated with pathological leukocyte counts and *agr* type 4, both of which could possibly serve as early indicators for high-risk infections. Inadequate initial therapy was administered to more than one quarter of patients and associated with decreased survival. Almost one third of patients were wrongly treated with second-line antibiotics in the first week of treatment. Increased disease severity was associated with the type of infection focus, *spa* clonal complex and enterotoxin genes *seg* and *sei*. Methicillin resistance occurred in 10.2% of isolates, of which 22.2% were LA-MRSA. We report a low rate of biofilm formation but high rate of nuclease activity among our bacteremia isolates. Clonality and prevalence of toxin genes were associated with several clinical and pathogen features. Overall, this study demonstrates a high burden of disease posed by SAB even in an advanced medical setting. It reveals many interesting correlations between patient characteristics, microbiological features, clinical presentation, and outcome of SAB and contributes to a better understanding of this frequent and severe disease.

## Figures and Tables

**Table 1:** Antibiotic substances defined as adequate.

First-Line Therapy	Second-Line Therapy
- 1st generation cephalosporins (e.g., cefazolin) - 2nd generation cephalosporins (e.g., cefuroxime) - anti-staphylococcal penicillins (e.g., oxacillin, flucloxacillin)	linezolid daptomycin glycopeptides (e.g., vancomycin, teicoplanin)

**Table 2:** Patient characteristics and clinical data.

Parameter	Data
Sex - Female/male, *n* (%)	74/104 (41.6/58.4)
Age in years - Median (IQR ^1^) - Mean (SD ^2^) - Range	63.0 (49.8–74.0) 58.7 (21.0) 0–94
Age in categories, *n* (%) - 0–18 years - 19–55 years - 56–70 years - >70 years	13 (7.3) 51 (28.7) 49 (27.5) 65 (36.5)
Infection focus, *n* (%) - Intravascular device - Abscess and soft tissue - Pneumonia - Endocarditis - Bone or joint - Bone or joint implant - Others - Unclear - Missing	71 (40.1) 23 (13.0) 22 (12.4) 16 (9.0) 11 (6.2) 10 (5.6) 5 (2.8) 19 (10.7) 1 (0.6)
Presence of fever ≥38 °C - Positive/negative, *n* (%) - Missing, *n*	112/44 (71.8/28.2) 22
CRP - Median (IQR) in mg/dL - Missing, *n*	13.6 (7.3–23.5) 12
CRP categories - Normal (≤0.5 mg/dL), *n* (%) - Elevated (>0.5 mg/dL), *n* (%) - Missing, *n*	7 (4.2) 159 (95.8) 12
Leukocyte count - Median (IQR) in × 10³/µL - Missing, *n*	10.2 (6.5–15.5) 6
Leukocyte categories - Lowered (<3.9 × 10³/µL), *n* (%) - Normal (3.9–10.9 × 10³/µL), *n* (%) - Elevated (>10.9 × 10³/µL), *n* (%) - Missing, *n*	20 (11.6) 75 (43.6) 77 (44.8) 6
Mortality, *n* (%) - Patient deaths overall - Patient deaths within first 30 days	43 (24.2) 26 (14.6)
- Median follow-up time in days (CI ^3^)	21.0 (16.5–25.5)
- Median overall survival in days (CI)	160.0 (75.2–244.8)
Initial antimicrobial therapy - Adequate/inadequate, *n* (%) - MSSA: adequate/inadequate, *n* (%) - MRSA: adequate/inadequate, *n* (%) - Second-line antibiotics, *n* (%) - of these: applied to MRSA/MSSA, *n* (%) - of these: information missing, *n* - Missing, *n*	107/38 (73.8/26.2) 96/36 (72.7/27.3) 10/2 (83.3/16.7) 54 (37.2) 10/43 (18.9/81.1) 1 33

^1^*IQR* interquartile range, ^2^ *SD* standard deviation, ^3^ *CI* 95% confidence interval.

**Table 3:** Isolate characteristics.

Parameter	Data
Colony size, *n* (%) - Normal phenotype (NP) - Small colony variant (SCV) phenotype - Mixed (NP and SCV)	174 (97.8) 1 (0.6) 3 (1.7)
Alpha-toxin, *n* (%) - Positive/negative	175/3 (98.3/1.7)
Beta-toxin, *n* (%) - Positive - Negative - Mixed	19 (10.7) 156 (87.6) 3 (1.7)
Delta-toxin, *n* (%) - Positive/negative	87/91 (48.9/51.1)
Biofilm in % of the positive control - Median (IQR) - Range	0.2 (−0.5–1.5) −1.9–53.8
Biofilm categories, *n* (%) - No biofilm formation - Low biofilm formation - Moderate biofilm formation - Strong biofilm formation	157 (88.2) 18 (10.1) 2 (1.1) 1 (0.6)
Nuclease activity in RFU - Median (IQR) - Range	528.6 (358.8–743.9) 97.8–2281.9
Methicillin resistance - MSSA/MRSA, *n* (%) - Missing, *n*	159/18 (89.8/10.2) 1
*agr* type - *agr* 1, *n* (%) *- agr* 2, *n* (%) *- agr* 3, *n* (%) - *agr* 4, *n* (%) - Negative for all *agr* types, *n* (%) - Missing, *n*	109 (61.6) 33 (18.6) 31 (17.5) 3 (1.7) 1 (0.6) 1
Prevalence of toxin genes *- sea, n* (%) *- seb*, *n* (%) *- sec*, *n* (%) *- sed, n* (%) *- see, n* (%) *- seg, n* (%) *- seh, n* (%) *- sei, n* (%) *- sej, n* (%) *- tst-1, n* (%) *- pvl, n* (%) *- hlg, n* (%) *- eta, n* (%) *- etb, n* (%) *- etd, n* (%) - Missing, *n*	13 (7.3) 6 (3.4) 37 (20.9) 10 (5.6) 2 (1.1) 103 (58.2) 9 (5.1) 104 (58.8) 10 (5.6) 23 (13.0) 2 (1.1) 176 (99.4) 2 (1.1) 0 (0) 3 (1.7) 1

**Table 4:** Population structure as identified by *spa* typing, BURP analysis, *mecA* and *agr* PCR.

*spa*-CC	Number of Isolates	*spa* Types	Methicillin Resistance	*agr* Type
084	38	t084 (10), t091 (19), t144, t360, t491, t2616, t4802, t7071, t11193, t12178, t18220	MSSA	*agr* 1 (20) *agr* 2 (16) *agr* 3 (1)
012	21	t012 (9), t018 (2), t021, t046, t090, t122 (2), t363, t726, t840, t964, t1654	MSSA	*agr* 3
015	19	t015, t050 (2), t069, t095, t102, t133, t230, t302, t550, t583, t728 (2), t1231, t1510, t2195, t4153, t5032, t18219	MSSA	*agr* 1
005/032	18	t005 (3), **t032** (6), t223, t310, t608, t612, **t1770**, t2816, **t5857**, **t12754**, t18079	9/18 MRSA	*agr* 1
068/008	11	t008 (5), **t068**, t068, t211, t2455, t7222, t18713	1/11 MRSA	*agr* 1
034/011	5	**t011** (2), **t034**, t571, **t2576**	4/5 MRSA	*agr* 1
864	4	t153, t166, t352, t864	MSSA	*agr* 3 (3) negative (1)
065	3	t040, t065, t6137	MSSA	*agr* 1
NF^1^ #1	4	t002 (3), t311	MSSA	*agr* 2
NF #2	4	t156 (2), t160 (2)	MSSA	*agr* 2
NF #3	3	**t003** (2), **t264**	MRSA	*agr* 2
NF #4	3	t267 (2), t692	MSSA	*agr* 1 (2) *agr* 3 (1)
NF #5	2	t127, t177	MSSA	*agr* 3
NF #6	2	t364, t493	MSSA	*agr* 1
singletons	29	t056 (2), t078, t092, t100, t131, t148 (2), t159, t189, t216, t280, t335, t351, t428, t845, t933, t1305, t1430, t2227, **t5488**, t8108 (2), t17517, t18076, t18218, t18622, t18636, t18712	1/29 MRSA	mixed
excluded	10	t026 (4), t643, t693, t748, t1050, t1991, t3625	MSSA	mixed

^1^*NF* no founder, i.e., related *spa* types clustered without an identified founder *spa* type. In the column “*spa* types”, the number of isolates assigned to each *spa* type is given in brackets if a *spa* type was found in more than one isolate. In the column “*agr* type”, the number of isolates assigned to each *agr* type is given in brackets if different *agr* types were found within one *spa*-CC. MRSA *spa* types are printed in **bold**, livestock-associated (LA)-MRSA *spa types* are underlined.

**Table 5:** Impact of patient age on overall survival.

	0–18 Years	19–55 Years	56–70 Years	>70 Years
Overall mortality in %	7.7	15.7	26.5	32.3
Median overall survival time in days (CI)	n.a. ^1^	422.0 (n.a.^1^)	297.0 (0.0–618.4)	75.0 (35.2–114.8)

^1^*n.a.* no information available: median survival is not computed if too many cases are censored.

**Table 6:** Impact of initial therapy on patient survival.

	Adequate Initial Therapy	Inadequate Initial Therapy	Log-Rank *p*
Overall mortality in %	18.7	34.2	0.041
Median overall survival time in days (CI)	139.0 (36.6–241.4)	106.0 (20.2–191.8)	
30-day mortality in %	10.3	26.3	0.010

**Table 7:** Impact of leukocyte counts on patient survival.

	Leukocytes Lowered	Leukocytes Normal	Leukocytes Elevated
Overall mortality in %	45.0	17.3	26.0
Median overall survival time in days (CI)	67.0 (40.5–93.5)	422.0 (49.5–794.5)	106.0 (62.7–149.3)

## Data Availability

The data presented in this study are available in the Supplementary Materials uploaded to t https://www.mdpi.com/article/10.3390/jcm10071371/s1: SPSS Dataset S1: Full dataset containing all analyzed parameters.

## Supplementary Materials

The following are available online at https://www.mdpi.com/article/10.3390/jcm10071371/s1, Table S1: Data on antibiotic therapy; Table S2: Results of *spa* typing / based upon repeat pattern (BURP) analysis.

## Appendix A

**Table A1:** Reference strains used in this study.

Strain	Used in	Reference
*S. aureus* RN4220	Delta-toxin assay	[14]
*S. aureus* RN6607	Delta-toxin assay	[14]
*S. aureus* Mu3	Delta-toxin assay	[14]
*S. epidermidis* RP62A	Biofilm assay	[97]
*S. carnosus* TM300	Biofilm assay	[97]
*S. aureus* AH1263 wt	Nuclease assay	[69]
*S. aureus* AH1680 (Δnuc1)	Nuclease assay	[69]

**Table A2:** Primers used for *spa* typing and single and multiplex PCRs.

Gene	Primer	Sequence (5′–3′)	Reference
*spa*	forward 1095F	AGACGATCCTTCGGTGAGC	[19]
	reverse 1517R	GCTTTTGCAATGTCATTTACTG	
*mecA*	mec-5 forward	AAAATCGATGGTAAAGGTTGGC	[21]
	mec-6 reverse	AGTTCTGCAGTACCGGATTTGC	
*agr*	agrSA-KON1 forward	ATGCACATGGTGCACATGC	[22]
	agrSA1-2 reverse	GTCACAAGTACTATAAGCTGCGAT	
	agrSA2-2 reverse	TATTACTAATTGAAAAGTGCCATAGC	
	agrSA3-2 reverse	GTAATGTAATAGCTTGTATAATAATACCCAG	
	agrSA4-2 reverse	CGATAATGCCGTAATACCCG	
*sea*	sea-3 forward	CCTTTGGAAACGGTTAAAACG	[23]
	sea-4 reverse	TCTGAACCTTCCCATCAAAAAC	
*seb*	seb-1 forward	TCGCATCAAACTGACAAACG	[23]
	seb-4 reverse	GCAGGTACTCTATAAGTGCCTGC	
*sec*	sec-3 forward	CTCAAGAACTAGACATAAAAGCTAGG	[23]
	sec-4 reverse	TCAAAATCGGATTAACATTATCC	
*sed*	sed-3 forward	CTAGTTTGGTAATATCTCCTTTAAACG	[23]
	sed-4 reverse	TTAATGCTATATCTTATAGGGTAAACATC	
*see*	see-2 forward	TAACTTACCGTGGACCCTTC	[23]
	see-3 reverse	CAGTACCTATAGATAAAGTTAAAACAAGC	
*seg*	seg-1 forward	AATGCTCAACCCGATCCTA	[24]
	seg-4 reverse	CTTCCTTCAACAGGTGGAGAC	
*seh*	seh-1 forward	TTAGAAATCAAGGTGATAGTGGC	[24]
	seh-2 reverse	TTTTGAATACCATCTACCCAAAC	
*sei*	sei-1 forward	GCCACTTTATCAGGACAATACTT	[24]
	sei-2 reverse	AAAACTTACAGGCAGTCCATCTC	
*sej*	sej-1 forward	CTCCCTGACGTTAACACTACTAATAA	[24]
	sej-2 reverse	TTGTCTGGATATTGACCTATAACATT	
*tst*	tst-3 foward	AAGCCCTTTGTTGCTTGCG	[23]
	tst-6 reverse	ATCGAACTTTGGCCCATACTTT	
*pvl*	pvl-1 forward	ATCATTAGGTAAAATGTCTGGACATGATCCA	[25]
	pvl-2 reverse	GCATCAASTGTATTGGATAGCAAAAGC	
*hlg*	hlg-1 forward	GCCAATCCGTTATTAGAAAATGC	[25]
	hlg-2 reverse	CCATAGAAGTAGCAACGGAT	
*eta*	eta-3 forward	CTAGTGCATTTGTTATTCAAGACG	[23]
	eta-4 reverse	TGCATTGACACCATAGTACTTATTC	
*etb*	etb-3 forward	ACGGCTATATACATTCAATTCAATG	[23]
	etb-4 reverse	AAAGTTATTCATTTAATGCACTGTCTC	
*etd*	etd-1 forward	AACTATCATGTATCAAGG	[26]
	etd-2 reverse	CAGAATTTCCCGACTCAG	
