# Supplementary material for: Correlations of Host and Bacterial Characteristics with Clinical Parameters and Survival in Staphylococcus aureus Bacteremia
Source: J Clin Med. 2021 Mar 28;10(7):1371. doi: 10.3390/jcm10071371 (PMC8037130; doi:10.3390/jcm10071371)
Supplement: Supplementary file 1 [file jcm-10-01371-s001.pdf]

Supplementary Material

Correlations of Host and Bacterial Characteristics with Clinical Parameters and Survival in *Staphylococcus aureus* Bacteremia

Hannah Wächter, Erdal Yörük, Karsten Becker, Dennis Görlich and Barbara C. Kahl

Supplementary Table S1. Data on antibiotic therapy.

Blood Culture No.	Blood Culture Date	MRSA	Available Data on Antibiotic Therapy
1118	12.05.2013	0	Tazobac/ Piperacillin 3* 4,5g 19.10.2014- 21.10.2014<br>Tazobac/ Piperacillin 2* 4,5 g 21.10.2014- 30.10.2014<br>Vancomycin 1* 250 mg 24.10.2014- 24.10.2014<br>Vancomycin 1* 250 mg 27.10.2014- 27.10.2014<br>Flucloxacillin 3* /d 20.11.2014- 21.11.2014<br>Cefuroxim 1* 1,5 g 21.11.2014- 22.11.2014<br>Cefuroxim 1* 750 mg from 22.11.2014 for 6 days<br>Cefuroxim 2* 250 mg 28.11.2014- 09.12.2014
1137	05.08.2013	0	Tazobac/ Piperacillin 3* 1g 03.08. - 06.08.2013
1138	01.08.2013	0	Meronem 3* 1 g from 01.08.2013- 14.08.2013
1140	05.08.2013	1	Tazobac/ Piperacillin 3* 4,5 g (no information on start date)
1141	06.08.2013	0	Vancomycin 2* 250 mg 07.08.2013- 08.08.2013<br>Vancomycin 2* 250 mg 11.08.2013- 12.08.2013<br>Gentamicin 3* 160 mg 07.08.2013- 17.08.2013<br>Gentamicin 3* 100 mg 19.08.2013- 04.09.2013<br>Rifampicin 1* 1200 mg 07.08.2013- 18.08.2013<br>Rifampicin 3* 300 mg 19.08.2013- 27.09.2013<br>Flucloxacillin 4* 3 g 08.08.2013- 18.08.2013<br>Flucloxacillin 4* 2 g 19.08.2013- 28.09.2013<br>Clindamycin 3* 900 mg 08.08.2013- 18.08.2013
1142	12.08.2013	0	Ceftriaxon 1* 2 g 2 days from 09.08.2013- 10.08.2013<br>Tazobac/ Piperacillin 3* 4,5 g 10.08.2013- 13.08.2013<br>Flucloxacillin 3* 3 g from 13.08.2013
1143	13.08.2013	0	Tazobac/ Piperacillin 3* 4,5 g 08.08.2013- 15.08.2013<br>Flucloxacillin 3* 2 g 15.08.2013- 20.08.2013<br>Rifampicin from 15.08.2013 for 16 days
1144	23.08.2013	0	Clarithromycin 2* 500 mg 24.08.- 25.08.2013<br>Tazobac/Piperacillin 3* 4,5 g 24.08.- 25.08.2013<br>Flucloxacillin 4* 2 g 25.08.2013- 06.09.2013<br>Gentamicin 1* 240 mg from 25.08.- 06.09.2013<br>Rifampicin 2* 600 mg from 06.09.2013<br>Cefuroxim 2* 500 mg from 06.09.2013
1145	26.08.2013	0	no information
1146	31.08.2013	0	Tazobac/ Piperacillin 3* 4,5 g 31.08.- 02.09.2013<br>Rifampicin 2* 300 mg from 02.09.2013<br>Gentamicin 3* 200 mg 02.09.2013- 07.09.2013<br>Gentamicin 1* 240 mg from 07.09.2013 for 17 days
1147	10.09.2013	0	Tazobac/ Piperacillin 3* 4,5 g 10.09. - 19.09.2013
1148	12.09.2013	0	Tazobac/ Piperacillin 3* 4,5 g 12.09. - 13.09.2013

			Flucloxacillin 3* 2 g 13.09.2013- 29.09.2013<br>Clindamycin 3* 300 mg 21.09.2013- 02.10.2013
1150	13.09.2013	0	Meronem 3* 1 g 12.09.2013 - 20.09.2013
1151	08.09.2013	0	Tazobac/ Piperacillin 3* 4,5 g 26.09. - 04.10.2013
1152	16.09.2013	0	Tazobac/ Piperacillin 3* 4,5 g from 16.09.2013<br>Clarithromycin 2* 500 mg from 16.09.2013
1153	19.09.2013	0	Flucloxacillin 4* 3 g 30.09.2013- 08.10.2013<br>Rifampicin 1* 600 mg 30.09.2013- 10.10.2013
1155	21.09.2013	1	Tazobac/ Piperacillin 3* 4,5 g 21.09. - 04.10.2013<br>Vancomycin 2* /d, 25.09.2013- 04.10.2013
1160	25.09.2013	0	Flucloxacillin for 14 days, start date unknown<br>Rifampicin for 14 days, start date unknown
1161	02.10.2013	0	no information
1162	07.10.2013	0	Ceftriaxon 1* 2 g 07.10.2013- 16.10.2013<br>Vancomycin 2* 1 g 08.10.2013- 11.10.2013
1164	10.10.2013	1	Tazobac/ Piperacillin<br>Gentamicin<br>Daptomycin<br>Fosfomycin
1165	14.10.2013	0	Fosfomycin 4* 5 g 04.11.2013- 06.11.2013<br>Meronem 3* 2 g 04.11.2013- 06.11.2013
1167	16.10.2013	0	Tazobac/ Piperacillin 3* 4,5 g 18.10. - 18.10.2013<br>Erythromycin 3* 100 mg 18.10.2013 - 30.10.2013<br>Ceftriaxon 1* 2 g 18.10.2013- 23.10.2013
1168	16.10.2013	0	no information
1170	20.10.2013	0	Gentamicin 1* 240 mg 19.10.2013- 21.10.2013<br>Vancomycin 2*/d 21.10.2013- 01.11.2013<br>Meronem 3*/d 22.10.2013- 01.11.2013<br>Cotrimoxazol 2* 960mg from 08.11.2013 for 7 days<br>Ciprofloxacin 2* 500 mg 21.11.2013- 29.11.2013<br>Penicillin 1*/d 21.11.2013- 29.11.2013
1171	20.10.2013	0	Tazobac/ Piperacillin 2* 4,5 g 20.10. - 24.10.2013<br>Vancomycin 2*/d 21.10.2013- 23.10.2013<br>Vancomycin 1*/d 23.10.2013- 28.10.2013<br>Meronem 2*/d 24.10.2013- 05.11.2013
1172	21.10.2013	0	Tazobac/ Piperacillin 3* 4,5 g 20.10. - 22.10.2014<br>Flucloxacillin 4* 2 g 22.10.2014- 01.11.2014<br>Cefuroxim 2* 250 mg 13.11.2014- 20.11.2014
1177	22.10.2013	0	Flucloxacillin from 26.10.2013<br>Rifampicin from 26.10.2013<br>Meronem from 26.10.2013
1178	25.10.2013	0	Ceftriaxon 1* 2 g 26.10.2013- 13.11.2013
1179	30.10.2013	0	Teicoplanin 2* 140 mg 01.11.2013- 05.11.2013<br>Teicoplanin 2* 150 mg 08.11.2013- 15.11.2013<br>Flucloxacillin 3* 450 mg 05.11.2013- 08.11.2013
1180	01.11.2013	0	Tazobac/ Piperacillin 3* 4,5 g 01.11. - 08.11.2013<br>Meronem 3* 1 g 08.11.2013- 21.11.2013
1181	04.11.2013	0	Tazobac/ Piperacillin 2* 4,5 g 04.11. - 05.11.2013
1182	06.11.2013	0	Tazobac/ Piperacillin 3* 4,5 g 07.11. - 08.11.2013<br>Flucloxacillin 4* 3 g from 08.11.2013 for 8 days
1183	06.11.2013	0	Clindamycin 3* 600 mg 05.11.2013- 11.12.2013<br>Ciprofloxacin 2* 400 mg 05.11.2013- 13.11.2013

			Amoxiclav 2,2g 3* 2,2 g 05.11.2013- 08.11.2013<br>Ampicillin 3* 4 g 09.11.2013- 11.12.2013<br>Combactam 3* 1 g 09.11.2013- 11.12.2013<br>Amoxiclav 3* 875/ 125 mg from 11.12.2013 for 7 d
1184	05.11.2013	0	Flucloxacillin 3* 2 g 10.11.2013- 14.11.2013<br>Rifampicin 2* 300 mg 10.11.2013- 27.11.2013<br>Flucloxacillin 3* 3 g 14.11.2013- 28.11.2013<br>Rifampicin 2* 450 mg 27.11.2013- 12.12.2013<br>Amoxiclav 875/ 125 mg 2*/d 28.11.- 29.11.2013<br>Amoxiclav 500/ 125 mg 2*/d 29.11.- 13.12.2013<br>Levofloxacin 1* 500 mg from 12.12.2013<br>Clindamycin 2* 600 mg from 12.12.2013
1185	09.11.2013	0	Cefuroxim 3* 1,5 g 11.11.2013- 12.11.2013<br>Rifampicin 2* 600 mg 12.11.2013- 14.11.2013<br>Rifampicin 2* 600 mg from 14.11.2013<br>Flucloxacillin 3* 2 g from 12.11.2013
1188	19.11.2013	0	Flucloxacillin 3* 3 g 21.11.2013- 04.12.2013<br>Clindamycin 2* 600 mg 21.11.2013- 02.12.2013<br>Cefaclor 3*/d from 05.12.2013
1189	17.11.2013	0	Ceftriaxon 1* 2 g 14.11.2013- 19.11.2013<br>Tazobac/ Piperacillin 3* 4,5 g 19.11. - 22.11.2013<br>Clarithromycin 2* 500 mg 19.11. - 26.11.2013<br>Cefuroxim 3* 750 mg from 22.11. - 22.11.2013<br>Cefuroxim 3* 1,5 g 22.11.2013- 29.11.2013
1195	24.11.2013	0	Tazobac/ Piperacillin 3* 4,5 g 23.11. - 26.11.2013<br>Gentamicin 1* 240 mg 23.11.2013- 25.11.2013<br>Vancomycin 2* 1 g 25.11.2013- 28.11.2013<br>Meronem 3*/d 26.11.2013- 05.12.2013<br>Linezolid 2* 600 mg 28.11.2013- 04.12.2013
1199	04.12.2013	0	Cefuroxim 3* 1,5 g from 03.12.2013 for 21 days
1200	05.12.2013	1	Imipenem+Cilastatin 500 mg 02.12. - 03.12.2013<br>Daptomycin, no further information<br>Linezolid, no further information<br>Meronem, no further information
1203	11.12.2013	0	Ciprofloxacin 2* 200 mg 12.12.2013- 13.12.2013<br>Augmentan (AmoxiClav) 3* 2,2g 12.12.- 13.12.13<br>Clindamycin 3* 600 mg 12.12.2013- 16.12.2013<br>Levofloxacin 2* 5 mg 14.12.2013- 16.12.2013<br>Cefuroxim 2* 750 mg 14.12.2013- 15.12.2013<br>Cefuroxim 2* 1,5 g 15.12.2013- 25.12.2013
1204	18.12.2013	0	Meronem 3* 1 g 18.12.2013- 28.12.2013<br>Vancomycin 2*/d 19.12.2013- 22.12.2013<br>Linezolid 2*/d 22.12.2013- 30.12.2013<br>Meronem 3*/d 10.01.2014- 23.01.2014<br>Vancomycin 2*/d 10.01.2014- 13.01.2014<br>Linezolid 2* 600 mg 13.01.2014- 22.01.2014<br>Tobramycin 1* 240 mg 13.01.2014- 20.01.2014
1205	23.12.2013	0	Fosfomycin 1* 3 g 23.12.2013- 23.12.2013<br>Meronem 3* 1 g 03.01.2014- 13.01.2014
1206	29.12.2013	0	Vancomycin for 31 days, no further information<br>Rifampicin for 31 days, no further information<br>Meronem for 31 days, no further information

1208	07.01.2014	0	Meronem, no further information<br>Vancomycin, no further information<br>Amoxiclav, no further information<br>Rifampicin 2* 300 mg 17.01.2014- 18.01.2014<br>Cefuroxim 3* 1,5 g 17.01.2014- 18.01.2014<br>Tazobac/ Piperacillin from 25.01.2014
1209	08.01.2014	0	Tazobac/ Piperacillin until 08.01.2015<br>Flucloxacillin 08.01.2014- 08.01.2014<br>Teicoplanin 09.01.2014- 14.01.2014<br>Cefuroxim 09.01.2014- 14.01.2014<br>Daptomycin from 14.01.2014<br>Ceftazidim 20.01.2014- 31.01.2014<br>Ciprofloxacin 20.01.2014- 31.01.2014<br>Teicoplanin 07.03.2014- 17.03.2014
1210	08.02.2014	0	Tazobac/ Piperacillin 3* 4,5 g 10.02. - 16.02.2014<br>Vancomycin 10.02.2014- 16.02.2014<br>Flucloxacillin 1* 2 g 11.02.2014- 11.02.2014
1211	13.02.2014	0	Tazobac/ Piperacillin for 13 days<br>Meronem for 13 days<br>Vancomycin for 13 days<br>Flucloxacillin for 13 days<br>Meronem for 13 days
1212	14.02.2014	0	no information
1213	16.02.2014	0	Flucloxacillin 3* 3 g from 22.02.2014 for 5 days<br>Clindamycin 3* 600 mg from 22.02.2014 for 5 days
1216	17.02.2014	0	Ceftriaxon 1* 4 g 17.02.2014- 18.02.2014<br>Gentamicin 1* 240 mg 17.02.2014- 19.02.2014<br>Vancomycin 3*/d 18.02.2014- 18.02.2014<br>Meronem 3* 2 g from 19.02.2014- 04.03.2014<br>Tazobac/ Piperacillin 3* 4 g 04.03. - 05.03.2014<br>Ciprofloxacin 2* 500 mg 05.03.2014- 12.03.2014<br>Augmentan (AmoxiClav) 3* 500 mg 05.03. - 12.03.2014<br>Tazobac/ Piperacillin 3* 4 g 12.03. - 17.03.2014<br>Gentamicin 1* 240 mg 12.03.2014- 14.03.2014<br>Vancomycin 3* 750 mg 15.03.2014- 21.03.2014<br>Meronem 3* 2 g 17.03.2014- 27.03.2014<br>Vancomycin 3* 650 mg 21.03.2014- 27.03.2014<br>Floxal (Ofloxacin) 3*/d
1218	19.02.2014	0	Flucloxacillin 4* 3 g 19.02.2014- 11.03.2014<br>Rifampicin 19.02.2014- 11.03.2014<br>Vancomycin 3* 500 mg from 11.03.2014 for 3 days<br>Linezolid 2* 600 mg for 10 days
1219	27.02.2014	0	Cotrim 240 mg/ 5 ml Sulfameth 2* 20ml for 1 day<br>Cotrimoxazol 2* 960 mg for 2 days<br>Vancomycin 2* 250 mg 28.02.2014- 28.02.2014<br>Gentamicin 2* 100 mg 28.02.2014- 18.03.2014<br>Rifampicin 2* 500 mg 28.02.2014- 11.04.2014<br>Flucloxacillin 3* 2 g 28.02.2014- 11.04.2014
1221	10.03.2014	0	Meronem 2* 1 g 11.03.2014- 27.03.2014<br>Amoxiclav 2* 0,5 g 18.03.2014- 27.03.2014
1222	14.03.2014	0	Flucloxacillin 3* 2 g 14.03.2014- 15.03.2014
1223	22.03.2014	0	Tazobac/ Piperacillin for 2 days, no further information

1224	22.03.2014	0	Ciprofloxacin 2* 500 mg 21.03.2014- 22.03.2014<br>Tazobac/ Piperacillin 3* 4,5 g 21.03. - 22.03.2014<br>Gentamicin 1* 240 mg 22.03.2014- 22.03.2014<br>Vancomycin 2* 1 g 22.03.2014- 02.04.2014<br>Meronem 3* 1 g 22.03.2014- 04.04.2014<br>Ciprofloxacin 2* 500 mg from 27.03. - 02.04.2014
1225	25.03.2014	1	Daptomycin 1* 500 mg 12.04.2014- 27.04.2014<br>Daptomycin 1* 500 mg 07.05.2014- 16.05.2014<br>Linezolid 2* 600 mg 16.05.2014- 10.06.2014<br>Daptomycin 1* 500 mg 25.06.2014- 29.07.2014<br>Tazobac/ Piperacillin 3* 4,5 g 26.06. - 29.07.2014<br>Ciprofloxacin 2* 250 mg 09.07.2014- 27.07.2014<br>Linezolid 2* 600 mg 29.07.2014- 20.08.2014
1228	25.03.2014	0	Tazobac/ Piperacillin 3* 4,5 g 25.03. - 27.03.2014<br>Flucloxacillin 3* 1 g 27.03.2014- 02.04.2014<br>Ciprofloxacin 2* 400 mg 29.03.2014- 04.04.2014
1230	27.03.2014	0	Tazobac/ Piperacillin 27.03.2014- 01.04.2014<br>Vancomycin 27.03.2014- 31.03.2014<br>Flucloxacillin 3* 2 g 28.03.2014-03.04.2014
1235	26.03.2014	0	Teicoplanin 2* 70 mg 26.03.2014- 27.03.2014<br>Vancomycin 3* 150 mg 27.03.2014- 29.03.2014<br>Meronem 3* 150 mg 27.03.2014- 01.04.2014
1237	12.04.2014	1	no information
1240	19.04.2014	1	Ceftriaxon 1* 2 g 18.04.2014- 19.04.2014<br>Clindamycin 3* 600 mg 18.04.2014- 05.06.2014<br>Tazobac/ Piperacillin 3* 4,5 g 19.04. - 05.06.2014<br>Ciprofloxacin 2* 400 mg 13.06.2014- 25.06.2014<br>Tazobac/ Piperacillin 3* 4,5 g 24.06. - 25.06.2014<br>Vancomycin 4* 250 mg 25.06.2014- 07.07.2014
1241	16.04.2014	0	Clindamycin 2* 600 mg, no further information
1243	22.04.2014	0	no information
1245	23.04.2014	0	Ciprofloxacin 2* 500 mg 04.04.2014- 22.04.2014<br>Tazobac/ Piperacillin 3* 4,5 g 22.04. - 26.04.2014<br>Gentamicin 1* 200 mg 22.04.2014- 26.04.2014<br>Meronem 3* 1 g 26.04.2014- 08.05.2014<br>Vancomycin 2* 1 g 26.04.2014- 05.05.2014<br>Cotrimoxazol 2* 960 mg from 11.05.2014<br>Linezolid 2* 600 mg 15.05.2014- 20.05.2014<br>Meronem 3* 1 g 15.05.2014- 04.06.2014<br>Rifampicin 3* 400 mg 22.05.2014- 16.06.2014<br>Tigecyclin 2* /d 22.05.- 18.06.2014<br>Ciprofloxacin 2* 200 mg 04.06.2014- 13.06.2014<br>Tazobac/ Piperacillin 1* 4,5 g 13.06. - 18.06.2014
1247	30.04.2014	0	Amoxiclav 3* 1 g from 28.04.2014<br>Tazobac/ Piperacillin 3* 4,5 g 30.04. - 06.05.2014<br>Klacid 2* 250 mg 30.04.2014- 06.05.2014<br>Gentamicin 3* 80 mg 06.05.2014- 20.05.2014<br>Flucloxacillin 3* 4 g 06.05.2014- 18.06.2014
1249	08.05.2014	0	Tazobac/ Piperacillin 3* 4,5 g 10.05. - 10.05.2014<br>Tazobac/ Piperacillin 3* 4,5 g 13.05. -18.05.2014<br>Cefuroxim 3* 750 mg 10.05.2014- 13.05.2014
1251	11.05.2014	0	Flucloxacillin, no further information

			Gentamicin, no further information<br>Rifampicin, no further information<br>Daptomycin, no further information<br>Meronem, no further information
1253	18.05.2014	0	Tazobac/ Piperacillin 2* 4,5 g
1256	18.05.2014	0	Meronem 2* 1 g 19.05.2014- 21.05.2014<br>Vancomycin 2* 1 g 19.05.2014- 21.05.2014<br>Flucloxacillin 3* 3 g from 20.05.2014- 26.05.2014
1258	23.05.2014	0	AmoxiClav 3* 2,2 g 31.05.2014- 28.06.2014<br>Cotrimoxazol 3* 1920 mg 31.05.- 02.06.2014<br>Cotrimoxazol 3* 960 mg 02.06.- 10.06.2014<br>Rifampicin 2* 300 mg 31.05.2014- 06.07.2014<br>AmoxiClav 2* 875 mg 28.06.2014- 06.07.2014<br>Ceftazidim 3* 2 g from 06.07.2014<br>Tobramycin 3* 80 mg 06.07.2014- 11.07.2014
1259	24.05.2014	0	Cefuroxim 2* 1,5 g 24.05.2014- 26.05.2014<br>Cefuroxim 3* 1,5 g 26.05.2014- 02.06.2014<br>Flucloxacillin 3* 4 g from 02.06.2014
1265	02.06.2014	0	Tazobac/ Piperacillin for 6 days, no further information
1266	04.06.2014	0	Tazobac/ Piperacillin 3* 4,5 g 03.06. - 04.06.2014<br>Ceftazidim 3* 2 g 04.06.2014- 04.06.2014<br>Cefuroxim 1* 1,5 g 04.06.2014- 06.06.2014<br>Clindamycin 3* 600 mg from 06.06.14 for 81 days<br>Meronem 3* /d from 06.06.2014 for 81 days
1267	06.06.2014	0	no information
1268	04.06.2014	0	Tazobac/ Piperacillin 2* 4,5 g 04.06. - 09.06.2014<br>Floxal (Ofloxacin) 3* /d 04.06.2014- 01.07.2014<br>Clindamycin 3* 600 mg 04.06.2014- 10.06.2014<br>Tazobac/ Piperacillin 3* 4,5 g 09.06. - 18.06.2014<br>Cotrimoxazol 2* 960 mg 13.06.2014-16.06.2014<br>Ciprofloxacin 2* 500 mg 18.06.2014- 20.06.2014<br>Tazobac/ Piperacillin 3* 4,5 g 20.06. - 01.07.2014<br>Ciprofloxacin 2* 500 mg 01.07.2014- 03.07.2014<br>Moxifloxacin 1* 400 mg from 03.07.2015
1269	16.06.2014	1	Vancomycin 1* 1 g 17.06.2014- 18.06.2014<br>Daptomycin 1* 350 mg 20.06.2014- 22.07.2014<br>Daptomycin 1* 500 mg 23.07.2014- 30.07.2014
1270	15.06.2014	1	no information
1271	15.06.2014	0	Vancomycin 1* 140 mg 16.06.2014- 16.06.2014<br>Vancomycin 1* 150 mg 17.06.2014- 18.06.2014
1274	20.06.2014	0	Ampicillin for 7 days<br>Gentamicin for 7 days<br>Tazobac/ Piperacillin from 20.06.2014<br>Vancomycin from 20.06.2014<br>Tazobac/ Piperacillin from 20.06.2014<br>Flucloxacillin from 20.06.2014<br>Tazobac/ Piperacillin 08.07.2014- 15.07.2014<br>Cefuroxim 18.07.2014- 30.07.2014<br>Cotrimoxazol, no further information
1275	20.06.2014	0	Flucloxacillin 3* 1 g from 23.06.2014<br>Ciprofloxacin 2* 500 mg 04.07.2014- 10.07.2014
1277	21.06.2014	0	Vancomycin 1* 750 mg 21.06.2014- 24.06.2014

			Floxal eye drops 5* /d 22.06. - 24.06.2014
1281	23.06.2014	0	Daptomycin 1* 500 mg 15.10.2014- 03.11.2014<br>Meronem 4* 1 g 15.10.2014- 03.11.2014
1284	26.06.2014	1	Ciprofloxacin 2* 400 mg 20.06.2014 - 26.06.2014<br>Tazobac/ Piperacillin 3* 4,5 g 26.06. - 08.07.2014<br>Gentamicin 1* 240 mg 26.06.2014- 27.06.2014<br>Linezolid 2* 600 mg 27.06.2014- 28.06.2014<br>Linezolid 3* 600 mg 28.06.2014- 01.07.2014<br>Linezolid 2* 600 mg from 01.07.2014
1285	25.06.2014	0	Cotrimoxazol 2* 960 mg 06.06.2014- 09.06.2014<br>Levofloxacin 2* 250 mg 06.06.2014- 11.06.2014<br>Tazobac/ Piperacillin 3* 4,5 g 10.06. - 19.06.2014<br>Tazobac/ Piperacillin 3* 4,5 g 25.06. - 26.06.2014<br>Gentamicin 1* 240 mg 25.06.2014 – 26.06.2014
1286	28.06.2014	0	Meronem 3* /d 28.06.2014- 10.07.2014<br>Vancomycin 1* 1 g 02.07.2014- 10.07.2014
1287	11.07.2014	0	Flucloxacillin 3* 800 mg 25.11.2013- 11.12.2013<br>Tazobac/ Piperacillin 4* 1,2 g 26.11. - 26.11.2013<br>Daptomycin 1* 150 mg 26.11.2013- 27.11.2013<br>Clindamycin 3* 300 mg 27.11.2013- 14.12.2013<br>Flucloxacillin 3* 800 mg 11.12.2013-14.12.2013
1288	10.07.2014	0	Clindamycin 3* 600 mg 08.07.2014- 10.07.2014<br>Flucloxacillin 3* 4 g 10.07.2014- 30.07.2014<br>Gentamicin 3* 80 mg 10.07.2014- 13.07.2014<br>Daptomycin 1* 350 mg 13.07.2014- 15.07.2014<br>Meronem 3* 1 g 13.07.2014- 29.07.2014<br>Fosfomycin 3* 4 g 15.07.2014- 21.07.2014<br>Rifampicin 3* 300 mg 21.07.2014- 28.07.2014<br>Rifampicin 2* 600 mg 28.07.2014-30.07.2014
1289	12.07.2014	0	Ceftriaxon 1* 2 g 12.07.2014- 13.07.2014<br>Cefuroxim 3* 1,5 g 17.07.2014- 23.07.2014<br>Rifampicin 2* 300 mg 17.07.2014- 23.07.2014
1291	14.07.2014	0	Ciprofloxacin 11.07.2014- 12.07.2014<br>Vancomycin 14.07.2014- 17.07.2014<br>Cefuroxim „high dosage“ 15.07.2014- 22.07.2014<br>Gentamicin 1* 240 mg 15.07.2014- 16.07.2014
1300	21.07.2014	0	Ciprofloxacin 21.07.2014- 21.07.2014<br>Tazobac/ Piperacillin 3* 4,5 g 22.07.- 31.07.2014<br>Vancomycin 2* /d 22.07.2014- 01.08.2014<br>Flucloxacillin 4* 1 Amp. from 18.04.2014<br>Gentamicin 3* 80 mg 01.08.2014- 04.08.2014<br>Gentamicin 80 / 40 / 80 mg 04.08. -13.08.2014<br>Rifampicin 2* 250 mg 02.08.2014-13.08.2014
1303	16.07.2014	0	Meronem 3* 1 g 17.07.2014- 23.07.2014<br>Cefuroxim 3* 1,5 g 17.07.2014- 23.07.2014
1304	21.07.2014	0	Tazobac/ Piperacillin 3* 4,5 g 21.07. - 24.07.2014<br>Clindamycin 4* 600 mg 23.07.2014- 04.08.2014<br>Flucloxacillin 3* 2 g 23.07.2014- 04.08.2014
1305	17.07.2014	0	Meronem 3* 2 g 17.07.2014 - 19.07.2014
1307	24.07.2014	0	Tazobac/ Piperacillin 3* 4,5 g 23.07. - 25.07.2014<br>Tazobac/ Piperacillin 3* 4,5 g 14.08. - 18.08.2014<br>Flucloxacillin 3* 1,5 g 25.07.2014- 14.08.2014

			Amoclav 500/ 125 mg 2* /d
1309	28.07.2014	0	Flucloxacillin 4* 1,5 g 30.07.2014 - 07.08.2014<br>Rifampicin 1* 600 mg 31.07.2014 - 07.08.2014
1312	03.08.2014	0	Moxifloxacin 1* 400 mg 03.08.2014- 06.08.2014<br>Flucloxacillin 4* 3 g 06.08.2014- 15.08.2014<br>Rifampicin 2* 450 mg 06.08.2014- 15.08.2014
1313	04.08.2014	0	Tazobac/ Piperacillin 3* 4,5 g 03.08. - 11.08.2014
1314	04.08.2014	0	Cefuroxim 06.08.2014- 12.08.2014<br>Tazobac/ Piperacillin 12.08.2014 - 18.08.2014<br>Flucloxacillin 3* 4 g 14.08.2014 - 29.08.2014<br>Gentamicin 14.08.2014- 23.08.2014<br>Rifampicin 20.08.2014- 28.08.2014
1315	08.08.2014	1	Linezolid 2* 600 mg 07.08.2014- 08.08.2014<br>Tazobac/ Piperacillin 3* 4,5 g 07.08. - 08.08.2014<br>Tazobac/ Piperacillin 2* 4,5 g 08.08. - 18.08.2014<br>Daptomycin 1* 700 mg 08.08.2014- 18.08.2014<br>Rifampicin 2* 450 mg 08.08.2014- 15.08.2014
1317	17.08.2014	0	Flucloxacillin 3* 4 g from 21.08.14 for 6 weeks<br>Gentamicin 1* 210 mg from 21.08.14 for 6 weeks
1323	29.08.2014	0	Rifampicin 2* 300 mg 03.09.2014 - 05.09.2014<br>Flucloxacillin 4* 4 g 03.09.2014 - 11.09.2014
1325	06.09.2014	0	Linezolid 2* 600 mg from 07.09.2014
1326	09.09.2014	0	Ciprofloxacin 2* 400 mg 10.09.2014- 10.09.2014<br>Clindamycin 4* 100 mg 10.09.2014- 05.10.2014<br>Vancomycin 3* 1 g 10.09.2014- 11.09.2014<br>Rifampicin 2* 450 mg 10.09.2014- 26.09.2014<br>Cefuroxim 4* 1,5 g 11.09.2014- 05.10.2014<br>Clindamycin 3* 100 mg 05.10.2014- 22.10.2014<br>Cefuroxim 3* 1,5 g 05.10.2014- 10.10.2014<br>Cefaclor 4* 1 g 10.10.2014- 22.10.2014
1327	12.09.2014	0	Clindamycin 3* 600 mg 11.09.2014- 13.09.2014
1328	13.09.2014	0	Augmentan 875/ 125 13.10.2014- 27.10.2014<br>Rifampicin 2* 600 bis 13.10.2014- 27.10.2014
1331	11.09.2014	0	Tazobac/ Piperacillin 3* /d 10.09. - 16.09.2014<br>Flucloxacillin 3* /d 16.09.2014 - 25.09.2014
1332	20.09.2014	0	Tazobac/ Piperacillin 3* 4,5 g, no further information
1333	21.09.2014	0	Rifampicin 2* 450 mg 23.09.2014- 20.10.2014<br>Flucloxacillin 3* 2 g 23.09.2014- 20.10.2014
1335	22.09.2014	0	Flucloxacillin 3* 1,5 g 24.09.2014 - 06.10.2014<br>Tazobac/ Piperacillin 3* 4,5 g 24.09. - 06.10.2014
1338	26.09.2014	0	Meronem for 2 days, no further information<br>Vancomycin for 2 days, no further information
1340	04.10.2014	0	no information
1342	20.10.2014	0	Meronem for 7 days, no further information<br>Cefuroxim for 14 days, no further information
1343	22.10.2014	1	Vancomycin 2* 250 mg 21.10.2014- 29.10.2014<br>Rifampicin 2* 500 mg 21.10.2014- 22.10.2014<br>Amoxicillin 3* 2 g 22.10.2014- 24.10.2014<br>Ampicillin 2g 3* 100 mg 24.10.2014- 30.10.2014<br>Rifampicin 2* 300 mg 25.10.2014- 20.11.2014<br>Ampicillin 3g 4* /d 30.10.2014- 30.11.2014

			Combactam 4* 250 mg 30.10.2014- 30.11.2014<br>Vancomycin 3* 250 mg 29.10.2014- 29.11.2014<br>Rifampicin 2* 450 mg 20.11.2014- 04.12.2014<br>Linezolid 2* 600 mg 29.11.2014- 06.12.2014
1344	31.10.2014	0	Amoxiclav 3* 1,2 g 02.11.2014- 08.11.2014<br>Erythromycin 2* 250 mg 12.11.2014- 12.11.2014
1345	02.11.2014	0	Cotrimoxazol 1* 960 mg 29.09.2014- 12.11.2014<br>Cotrimoxazol 1* 480 mg 08.11.2014- 10.11.2014<br>Cotrimoxazol 1* 480 mg from 12.11.2014<br>Amoxicillin 3* 750 mg 08.11.2014- 12.11.2014
1346	11.11.2014	0	Tazobac/ Piperacillin 3* 4,5 g 10.11. - 16.11.2014
1347	21.11.2014	0	Tazobac/ Piperacillin 3* 4,5 g 22.11.- 22.11.2014<br>Vancomycin 1* 500 mg 22.11.2014- 24.11.2014<br>Flucloxacillin 4* 3 g for 12 days, no further information
1349	25.11.2014	0	Ceftriaxon 1* 2 g 25.11.2014- 28.11.2014<br>Cefuroxim 2* 200 mg for 7 days, no further information
1350	27.11.2014	0	Amoxiclav 2* 2,2 g 27.11.2014- 28.11.2014<br>Vancomycin 2* 1 g 28.11.2014- 02.12.2014<br>Flucloxacillin 4* 2 g 28.11.2014- 02.12.2014<br>Ciprofloxacin 2* 750 mg from 03.12.2014
1351	26.11.2014	1	Clindamycin 3* 600 mg 26.11.2014- 27.11.2014<br>Tazobac/ Piperacillin 3* 4,5 g 26.11. - 01.12.2014<br>Ceftazidim 3* 2 g 26.11.2014- 28.11.2014<br>Flucloxacillin 5* 2 g 28.11.2014- 28.11.2014<br>Linezolid 2* 600 mg 28.11.2014- 03.12.2014<br>Meronem 3* 1 g 01.12.2014- 08.12.2014
1354	28.11.2014	0	Tazobac/ Piperacillin 27.11.2014- 30.11.2014<br>Vancomycin 27.11.2014- 30.11.2014<br>Flucloxacillin 3* 4 g 30.11.2014- 14.12.2014
1355	29.11.2014	0	no information
1360	14.12.2014	0	Ciprofloxacin 3* 500 mg 14.12.2014- 15.12.2014<br>Clindamycin 2* 600 mg 14.12.2014- 17.12.2014<br>Cefuroxim 3* 1,5 g 14.12.2014- 17.12.2014<br>Flucloxacillin 4* 2 g for 12 days
1361	16.12.2014	0	Tazobac/ Piperacillin 3* 4,5 g 13.12.- 17.12.2014
1362	20.12.2014	0	Tazobac/ Piperacillin 3* 4,5 g 21.12. - 23.12.2014<br>Flucloxacillin 4* 2 g 23.12.2014- 26.12.2014<br>Gentamicin 3* 80 mg 24.12.2014- 26.12.2014<br>Gentamicin 2* 80 mg 26.12.2014- 26.12.2014
1364	21.12.2014	0	Cefuroxim 3* 1,5 g 23.12.2014- 01.01.2015<br>Ceftriaxon 1* 2 g for 3 days
1365	30.12.2014	1	Clindamycin 3* 600 mg 30.12.2014- 31.12.2014<br>Ciprofloxacin 2* 400 mg 31.12.2014- 01.01.2015<br>Vancomycin 3* /d 01.01.2015- 18.01.2015<br>Gentamicin 2* 160 mg 02.01.2015- 07.01.2015
1368	03.01.2015	1	Vancomycin 2* 500 mg 04.01.2015- 08.01.2015
1369	03.01.2015	0	no information
1370	05.01.2015	0	Vancomycin 3* 740 mg 05.01.2015- 07.01.2015<br>Meronem 3* 920 mg 05.01.2015- 07.01.2015<br>Flucloxacillin 3* 500 mg 07.01.2015- 20.01.2015<br>Meronem 3* 920 mg 11.01.2015- 17.01.2015
1373	11.01.2015	0	Ceftriaxon 1* 2 g 10.01.2015- 14.01.2015

			Clindamycin 3* 600 mg 10.01.2015- 26.01.2015<br>Flucloxacillin 3* 1 g 10.01.2015- 23.01.2015<br>Flucloxacillin 3* 3 g 14.01.2015- 26.01.2015
1374	12.01.2015	0	Ceftazidim 12.01.2015- 21.01.2015<br>Gentamicin 14.01.2015- 16.01.2015
1375	20.01.2015	0	no information
1376	28.01.2015	0	Cefuroxim 3* 1,5 g 28.01.2015- 29.01.2015<br>Flucloxacillin 3* 2 g 29.01.2015- 30.01.2015<br>Flucloxacillin 3* 1 g 30.01.2015- 06.02.2015
1378	30.01.2015	0	Tazobac/ Piperacillin 3* 4,5 g 24.01.- 26.01.2015<br>Flucloxacillin 3* 1 g 26.01.2015- 30.01.2015<br>Flucloxacillin 3* 3 g 02.02.2015- 06.02.2015<br>Meronem 3* /d 30.01.2015- 02.02.2015
1379	30.01.2015	0	Meronem 3* 1 g for 22 days, no further information
1382	11.02.2015	0	Flucloxacillin 3* 1 g 12.02.2015- 05.03.2015<br>Gentamicin 2* 75 mg 12.02.2015- 17.02.2015<br>Gentamicin 2* 100 mg 17.02.2015- 19.02.2015<br>Gentamicin 3* 80 mg 19.02.2015- 05.03.2015<br>Rifampicin 2* 600 mg 22.02.2015- 24.02.2015
1383	17.02.2015	0	Flucloxacillin 3* 4 g from 21.02.2015 for 5 days
1384	20.02.2015	0	Ceftriaxon 1* 2 g 20.02.2015- 21.02.2015<br>Vancomycin 2* 1 g 20.02.2015- 21.02.2015<br>Flucloxacillin 6* 2 g 21.02.2015- 21.02.2015<br>Gentamicin 3* 70 mg 21.02.2015- 06.03.2015
1386	06.03.2015	1	Vancomycin 1 g local from 20.03.2015 for 20 days<br>Rifampicin 20.03.2015- 22.03.2015<br>Linezolid 23.03.2015- 05.04.2015
1387	14.03.2015	0	Ceftriaxon 1* 2 g 14.03.2015- 15.03.2015<br>Meronem 3* 1 g 15.03.2015- 16.03.2015<br>Vancomycin 2* 1 g from 15.03.2015- 16.03.2015<br>Tazobac/ Piperacillin 3* 4,5 g 20.03. - 27.03.2015
1388	18.03.2015	0	Tazobac/ Piperacillin 06.02.2015- 18.02.2015<br>Meronem, no further information<br>Ciprofloxacin, no further information
1389	18.03.2015	0	Flucloxacillin 4* 3 g 21.03.2015- 25.03.2015<br>Meronem 3* 1 g 21.03.2015- 25.03.2015
1390	20.03.2015	1	Augmentan 3* 2,2 g 21.03.2015- 24.06.2015<br>Rifampicin 2* 450 mg 21.03.2015- 11.05.2015<br>Vancomycin 2* 1 g 21.03.2015- 06.05.2015<br>Vancomycin 3* 1 g 06.05.2015- 06.06.2015<br>Vancomycin 3* 1,5 g 06.06.2015- 24.06.2014
1392	22.03.2015	0	Tazobac/ Piperacillin, no further information<br>Flucloxacillin, no further information<br>Clindamycin, no further information<br>Daptomycin, no further information
1396	23.03.2015	0	Tazobac/ Piperacillin 3* 4,5 g 07.03. - 13.03.2015<br>Ceftriaxon 1* 2 g 23.03.2015- 24.03.2015<br>Vancomycin 2* 1 g 24.03.2015- 07.04.2015<br>Meronem 2* 1 g 28.03.2015- 02.04.2015<br>Meronem 3* 1 g 02.04.2015- 11.04.2015
1398	08.04.2015	0	Ciprofloxacin 2* 500 mg 03.04.2015- 07.04.2015<br>Meronem 3* 500 mg 08.04.2015- 09.04.2015

			Vancomycin 2* 1 g 09.04.2015- 15.04.2015<br>Vancomycin 1* 500 mg 15.04.2015- 20.04.2015
1399	13.04.2015	0	Cotrimoxazol for 6 days, no further information
1400	17.04.2015	0	Meronem 15.04.2015- 15.05.2015<br>Vancomycin 2* 1 g 18.04.2015- 21.04.2015<br>Tazobac/ Piperacillin 3* 4,5 g 18.04. - 18.04.2015<br>Rifampicin 2* 450 mg 18.04.2015- 16.05.2015<br>Cefuroxim 4* 1,5 g 21.04.2015- 24.04.2015<br>Cefuroxim 4* 1,5 g 24.04.2015-16.05.2015<br>Clindamycin 3* 600 mg 26.04.2015- 29.04.2015
1401	19.04.2015	0	Ceftriaxon 1* 2 g 20.04.2015- 21.04.2015<br>Flucloxacillin 4* 3 g 21.04.2015- 27.04.2015<br>Moxifloxacin 2* 400 mg 29.04.2015- 02.05.2015<br>Flucloxacillin 3* 2 g 02.05.2015- 13.05.2015<br>Rifampicin 2* 300 mg 02.05.2015- 13.05.2015<br>Meronem 3* 1 g 06.05.2015- 13.05.2015
1404	22.04.2015	0	Cefuroxim 4* 1,5 g 25.04.2015- 28.04.2015<br>Fosfomycin 3* 5 mg 25.04.2015- 28.04.2015<br>Rifampicin 2* 500 mg 28.04.2015- 20.05.2015<br>Flucloxacillin 3* 1 g 28.04.2015- 20.05.2015<br>Flucloxacillin 4* 1 g 20.05.2015- 05.06.2015
1405	26.04.2015	0	Tazobac/ Piperacillin 2*1Amp. 13.05.- 19.05.2015<br>Flucloxacillin, no further information
1411	16.05.2015	0	Tazobac/ Piperacillin 3* 4,5 g 16.05.- 25.05.2015<br>Flucloxacillin 3* 4 g 18.05.2015- 21.05.2015<br>Flucloxacillin 3* 1 g 23.05.2015- 24.05.2015
1412	18.05.2015	0	Imipenem+Cilastatin 3*500mg 18.05.- 08.06.2015<br>Daptomycin 1* 500 mg 18.05.2015- 08.06.2015<br>Augmentan 2* 960 mg 08.06.2015-13.06.2015
1414	19.05.2015	0	Ceftriaxon 1* 2 g 19.05.2015- 21.05.2015<br>Clindamycin 3* 600 mg 19.05.2015- 21.05.2015
1415	23.05.2015	0	Tazobac/ Piperacillin 27.03.2015- 24.04.2015<br>Vancomycin for 2 days, no further information<br>Flucloxacillin for 2 days, no further information
1417	23.05.2015	0	Clindamycin 4* 600 mg 24.05.2015- 31.05.2015<br>Tazobac/ Piperacillin 3* 4,5 g 24.05.- 09.06.2015<br>Clindamycin 4* 600 mg 9 31.05.2015- 09.06.2015
1418	30.05.2015	0	Amoxicillin 3* 1 g 30.05.2015- 31.05.2015<br>Tazobac/ Piperacillin 3* 4,5 g 31.05. - 31.05.2015<br>Cefuroxim 3* 1,5 g 31.05.2015- 04.06.2015
1419	09.06.2015	0	Meronem 3* 1 g 09.06.2015- 11.06.2015<br>Linezolid 2* 600 mg 09.06.2015- 11.06.2015<br>Cotrimoxazol 2* 960 mg 11.06.2015- 13.07.2015<br>Flucloxacillin 3* 4 g 11.06.2015- 16.06.2015<br>Ciprofloxacin 2* 400 mg 17.06.2015- 23.06.2015<br>Cefuroxim 2* 1,5 g 25.06.2015- 28.06.2015<br>Tazobac/ Piperacillin 3* 4,5 g 11.07. - 13.07.2015<br>Ceftazidim 3* 2 g 11.07.2015- 14.07.2015<br>Tazobac/ Piperacillin 2* 4,5 g 13.07. - 20.07.2015<br>Ceftazidim 2* 1 g 14.07.2015- 20.07.2015<br>Tazobac/ Piperacillin 3* 4,5 g 24.07. - 03.08.2015<br>Vancomycin 2* 1 g 26.07.2015- 01.08.2015

			Meronem 3* 1 g 03.08.2015- 26.08.2015<br>Vancomycin 2* /d 03.08.2015- 05.08.2015<br>Vancomycin 1* /d 05.08.2015- 06.08.2015<br>Linezolid 2* 600 mg 06.08.2015- 26.08.2015
1420	09.06.2015	0	Flucloxacillin 09.06.2015- 15.06.2015<br>Meronem 22.06.2015- 29.06.2015<br>Augmentan 02.07.2015- 06.07.2015<br>Flucloxacillin 06.07.2015- 15.07.2015
1423	10.06.2015	0	Ciprofloxacin 2* 500 mg 26.05.2015- 05.06.2015<br>Ciprofloxacin 2* 400 mg 05.06.2015- 10.06.2015<br>Ceftazidim 3* 2 g 10.06.2015- 15.06.2015<br>Gentamicin 1* 240 mg 10.06.2015- 15.06.2015<br>Gentamicin 4* 240 mg 11.06.2015- 11.06.2015<br>Cotrimoxazol 2* 960 mg 16.06.2015- 22.06.2015
1425	04.06.2015	0	Tazobac/ Piperacillin 3* 4,5 g 04.06. - 04.06.2015<br>Amoxiclav 875/ 125 mg 05.06.2015- 14.06.2015
1426	05.06.2015	1	Tazobac/ Piperacillin 3* 4,5 g 05.06. - 07.06.2015<br>Clarithromycin 05.06.2015- 07.06.2015<br>Vancomycin 08.06.2015- 17.06.2015
1428	14.06.2015	0	Vancomycin 14.06.2015- 24.06.2015<br>Tazobac/ Piperacillin 14.06.2015- 24.06.2015<br>Meronem 15.06.2015- 26.06.2015<br>Cefuroxim 18.06.2015- 02.07.2015<br>Tazobac/ Piperacillin 3* 1,1 g 23.06. - 29.06.2015<br>Vancomycin 4* 180 mg 23.06.2015- 29.06.2015
1429	18.06.2015	0	Tazobac/ Piperacillin 3* 4,5 g 18.06. - 19.06.2015<br>Vancomycin 2* 1 g 19.06.2015- 23.06.2015<br>Rifampicin 2* 600 mg 21.07.2015- 22.07.2015<br>Flucloxacillin 4* 3 g from 21.07.2015, no further information<br>Daptomycin 1* 500 mg from 22.07.2015, no further information
1430	24.06.2015	0	Flucloxacillin 3* /d 25.06.2015- 29.06.2015<br>Flucloxacillin 3* /d 29.06.2015-03.07.2015<br>Rifampicin 1* 600 mg 25.06.2015- 03.07.2015
1431	23.06.2015	0	Cefuroxim 3* 1,5 mg 21.06.2015- 24.06.2015
1434	03.07.2015	0	Clindamycin 3* 600 mg 07.07.2015- 21.07.2015
1436	06.07.2015	0	Ceftriaxon for 2 days, no further information<br>Ampicillin for 2 days, no further information
1437	07.07.2015	0	Moxifloxacin 1* /d 07.07.2015- 08.07.2015<br>Tazobac/ Piperacillin 3* 4,5 g 08.07. - 16.07.2015
1438	16.07.2015	0	no information
1439	17.07.2015	0	Cefuroxim 3* 1,5 g 18.07.2015- 20.07.2015<br>Flucloxacillin 4* /d 20.07.2015- 03.08.2015<br>Rifampicin 2* 300 mg 20.07.2015- 04.08.2015
1441	22.07.2015	0	Meronem for 4 days, no further information<br>Linezolid danach for 4 days, no further information
1444	28.07.2015	0	no information
1410-1	05.05.2015		Vancomycin 3* 100 mg 06.05.2015- 08.05.2015<br>Vancomycin 3* 400 mg 08.05.2015- 10.05.2015<br>Vancomycin 4* 350 mg 10.05.2015- 12.05.2015<br>Flucloxacillin 3* 400 mg 12.05.2015- 19.05.2015<br>Flucloxacillin 4* 5 g from 19.05.2015

In the column “MRSA”, “0” represents an MSSA isolate and “1” encodes an MRSA isolate.

Supplementary Table S2. Results of *spa* typing / based upon repeat pattern (BURP) analysis.

spa Type	Repeat Succession	*n*	% of All 178 Isolates	BURP Result
t084	07-23-12-34-34-12-12-23-02-12-23	10	5.6	*spa*-CC084
t091	07-23-21-17-34-12-23-02-12-23	19	10.7	*spa*-CC084
t144	07-23-12-34-34-12-12-23-02-02-12-23	1	0.6	*spa*-CC084
t360	07-23-12-34-12-23-02-12-23	1	0.6	*spa*-CC084
t491	26-23-12-34-34-12-12-23-02-12-23	1	0.6	*spa*-CC084
t2616	07-23-21-17-34-12-23-02-12-23-02-12-23	1	0.6	*spa*-CC084
t4802	07-23-12-34-34-12-12-23-23-02-12-23	1	0.6	*spa*-CC084
t7071	07-23-20-12-34-34-12-23-02-12-23	1	0.6	*spa*-CC084
t11193	07-23-21-17-34-12-16-23-02-12-23	1	0.6	*spa*-CC084
t12178	07-23-12-34-34-12-12-16-02-12-23	1	0.6	*spa*-CC084
t18220	14-23-12-34-34-12-23-02-12-23	1	0.6	*spa*-CC084
t012	15-12-16-02-16-02-25-17-24-24	9	5.1	*spa*-CC012
t018	15-12-16-02-16-02-25-17-24-24-24	2	1.1	*spa*-CC012
t021	15-12-16-02-16-02-25-17-24	1	0.6	*spa*-CC012
t046	08-16-02-16-02-25-17-24-24-24	1	0.6	*spa*-CC012
t090	15-12-16-16-02-16-02-25-17-24-24-24	1	0.6	*spa*-CC012
t122	08-16-02-16-02-25-17-24-24	2	1.1	*spa*-CC012
t363	15-16-02-25-17-24	1	0.6	*spa*-CC012
t726	15-12-02-16-02-25-17-24	1	0.6	*spa*-CC012
t840	15-12-02-16-02-25-17-24-24	1	0.6	*spa*-CC012
t964	08-16-16-02-16-02-25-17-24	1	0.6	*spa*-CC012
t1654	15-12-16-02-16-02-25-17-17-24-24	1	0.6	*spa*-CC012
t015	08-16-02-16-34-13-17-34-16-34	1	0.6	*spa*-CC015
t050	08-16-02-16-34-34-17-34-16-34	2	1.1	*spa*-CC015
t069	08-16-02-16-34-13-17-34-16-16-34	1	0.6	*spa*-CC015
t095	08-16-02-16-34-34	1	0.6	*spa*-CC015
t102	08-16-13-17-34-16-34	1	0.6	*spa*-CC015
t133	08-16-02-16-34-16-34	1	0.6	*spa*-CC015
t230	08-16-02-16-34	1	0.6	*spa*-CC015
t302	08-16-02-16-34-13-17-13-16-34	1	0.6	*spa*-CC015
t550	08-17-34-16-34	1	0.6	*spa*-CC015
t583	08-16-02-16-34-13-16-34	1	0.6	*spa*-CC015
t728	08-16-34-16-34	2	1.1	*spa*-CC015
t1231	08-16-02-16-34-13-17-34-16-34-16-34	1	0.6	*spa*-CC015
t1510	08-16-02-16-34-13-17-34-16-13	1	0.6	*spa*-CC015
t2195	07-17-34-16-34	1	0.6	*spa*-CC015
t4153	08-16-02-16-34-80-17-34-16-34-34	1	0.6	*spa*-CC015
t5032	08-16-02-16-13-17-34-34	1	0.6	*spa*-CC015
t18219	08-23-16-34-13-16-34	1	0.6	*spa*-CC015
t005	26-23-13-23-31-05-17-25-17-25-16-28	3	1.7	*spa*-CC005/032
t032	26-23-23-13-23-31-29-17-31-29-17-25-17-25-16-28	6	3.4	*spa*-CC005/032
t223	26-23-13-23-05-17-25-17-25-16-28	1	0.6	*spa*-CC005/032
t310	26-23-31-05-17-25-17-25-16-28	1	0.6	*spa*-CC005/032
t608	26-23-31-29-17-25-17-25-16-28	1	0.6	*spa*-CC005/032
t612	26-23-23-13-16-31-29-17-31-29-17-25-17-25-16-28	1	0.6	*spa*-CC005/032
t1770	26-23-23-13-23-31-29-17-29-17-25-17-25-16-28	1	0.6	*spa*-CC005/032
t2816	26-23-13-23-31-31-05-17-25-17-25-16-28	1	0.6	*spa*-CC005/032

t5857	26-23-23-13-29-17-25-17-25-16-28	1	0.6	*spa*-CC005/032
t12754	26-23-23-13-23-23-29-17-31-29-17-25-17-25-16-28	1	0.6	*spa*-CC005/032
t18079	26-23-31-05-05-17-25-17-25-17-25-16-28	1	0.6	*spa*-CC005/032
t008	11-19-12-21-17-34-24-34-22-25	5	2.8	*spa*-CC068/008
t068	11-19-19-12-21-17-34-24-34-22-25	2	1.1	*spa*-CC068/008
t211	11-19-12-12-21-17-34-24-34-22-25	1	0.6	*spa*-CC068/008
t2455	11-12-21-21-17-34-24-34-22-25	1	0.6	*spa*-CC068/008
t7222	11-19-21-12-12-17-34-24-34-22-25	1	0.6	*spa*-CC068/008
t18713	11-19-19-12-12-17-34-24-34-22-25	1	0.6	*spa*-CC068/008
t011	08-16-02-25-34-24-25	2	1.1	*spa*-CC034/011
t034	08-16-02-25-02-25-34-24-25	1	0.6	*spa*-CC034/011
t571	08-16-02-25-02-25-34-25	1	0.6	*spa*-CC034/011
t2576	08-12-16-02-25-34-24-25	1	0.6	*spa*-CC034/011
t153	04-44-33-31-12-16-34-16-12-33-34	1	0.6	*spa*-CC864
t166	04-44-33-31-12-16-34-16-12-25-22-34	1	0.6	*spa*-CC864
t352	04-54-31-12-16-34-16-12-25-22-34	1	0.6	*spa*-CC864
t864	04-44-33-12-16-34-16-12-25-22-34	1	0.6	*spa*-CC864
t040	09-02-16-13-17-34-16-34	1	0.6	*spa*-CC065
t065	09-02-16-34-13-17-34-16-34	1	0.6	*spa*-CC065
t6137	09-02-16-34-13-17-34-16	1	0.6	*spa*-CC065
t002	26-23-17-34-17-20-17-12-17-16	3	1.7	clustered without founder
t311	26-23-17-34-20-17-12-17-16	1	0.6	
t156	07-23-12-33-22-17	2	1.1	clustered without founder
t160	07-23-21-24-33-22-17	2	1.1	
t127	07-23-21-16-34-33-13	1	0.6	clustered without founder
t177	26-23-21-16-34-33-13	1	0.6	
t364	04-34-17-32-17-23-24	1	0.6	clustered without founder
t493	04-34-17-66-32-17-23-24	1	0.6	
t003	26-17-20-17-12-17-17-16	2	1.1	clustered without founder
t264	26-17-20-17-17-17-16	1	0.6	
t267	07-23-12-21-17-34-34-34-33-34	2	1.1	clustered without founder
t692	07-12-21-17-34-34-34-34-33-34	1	0.6	
t056	04-20-12-17-20-17-12-17-17	2	1.1	singleton
t078	04-21-12-41-20-17-12-12-17	1	0.6	singleton
t092	26-23-20-31-17-25-17-25-16-28	1	0.6	singleton
t100	26-23-02-12-23-02-34-34	1	0.6	singleton
t131	07-23-12-34-33-34	1	0.6	singleton
t148	07-23-12-21-12-17-20-17-12-12-17	2	1.1	singleton
t159	14-44-13-12-17-17-23-18-17	1	0.6	singleton
t189	07-23-12-21-17-34	1	0.6	singleton
t216	04-20-17-20-17-31-16-34	1	0.6	singleton
t280	04-20-17-12-12-17	1	0.6	singleton
t335	07-23-12-34-34-12-23	1	0.6	singleton
t351	11-19-12-21-22-25	1	0.6	singleton
t428	08-12-16-34-02-43-34-16-16-02-17-16	1	0.6	singleton
t845	26-23-13-16-28	1	0.6	singleton
t933	08-16-16-02-25-51-68-02-24-02-24	1	0.6	singleton
t1305	26-23-17-34-17-20-16	1	0.6	singleton
t1430	07-16-23-02-12-23-02-34	1	0.6	singleton
t2227	07-23-12-21-17-34-34-24-24-33-34	1	0.6	singleton
t5488	09-02-25-34-24-25	1	0.6	singleton

t8108	26-23-13-23-31-29-17-31-29-17-31-29-16-17-25-16-28	2	1.1	singleton
t17517	09-34-13-17-34-16-13	1	0.6	singleton
t18076	09-34-34-13-34-16-34	1	0.6	singleton
t18218	04-12-41-17-12-12-17	1	0.6	singleton
t18622	15-12-16-02-16-02-25-17-24-24-17-24-24-17-24-24	1	0.6	singleton
t18636	121-21-16-34-17-82-24-17-17	1	0.6	singleton
t18712	04-44-33-31-12-16-34-34-16-12-25-16-22-22-34	1	0.6	singleton
t026	08-16-34	4	2.2	excluded
t643	04-21-12-17	1	0.6	excluded
t693	07	1	0.6	excluded
t748	15-12-17-24	1	0.6	excluded
t1050	08-96-34	1	0.6	excluded
t1991	08-17	1	0.6	excluded
t3625	08-16-34-25	1	0.6	excluded
non-typable	-	2	1.1	not analyzed

spa types are sorted by frequency of their respective *spa*-CCs. For related *spa* types that were clustered without a founder, the cells in the column "BURP analysis" were merged to show which *spa* types are related to each other. By default settings, *spa* types were grouped if cost was less than or equal to four and *spa* types shorter than five repeats were excluded from the analysis.
